# Efficacy of transcranial direct current stimulation for disorders of consciousness: an updated systematic review and meta-analysis based on sham-controlled randomized trials

**DOI:** 10.3389/fnins.2026.1918788

**Published:** 2026-08-04

**Authors:** Fuqiang Wang, Yaojiang Li, Lixia Deng, Congping Huang, Liangxiang Zhang, Chunmei Zhao, Haoqiang He, Xiao Lu, Yunhong Deng

**Affiliations:** 1 Department of Rehabilitation Medicine, Guangdong Sanjiu Brain Hospital, Guangzhou, Guangdong, China; 2 Department of Rehabilitation Therapy, Guangdong Sanjiu Brain Hospital, Guangzhou, Guangdong, China

**Keywords:** Coma Recovery Scale–Revised, disorders of consciousness, meta-analysis, neuromodulation, transcranial direct current stimulation

## Abstract

**Background:**

Transcranial direct current stimulation (tDCS) may improve behavioral signs of consciousness in patients with disorders of consciousness (DoC), but evidence from randomized controlled trials (RCTs) and previous meta-analyses has remained inconsistent. We evaluated the efficacy and safety of tDCS in patients with DoC and explored potential effect modifiers.

**Methods:**

PubMed, Embase, Scopus, Web of Science, and the Cochrane Library were searched for RCTs comparing active tDCS with sham stimulation. For crossover trials, only first-period data were extracted. The primary outcome was the change in Coma Recovery Scale–Revised (CRS-R) score. Mean differences (MDs) with 95% confidence intervals (CIs) were pooled across studies. Subgroup analyses were performed according to baseline level of consciousness, etiology, disease duration, stimulation target, and total stimulation dose.

**Results:**

Fourteen trials including 330 patients were included. Active tDCS was associated with greater improvement in CRS-R change scores than sham stimulation (MD = 1.02, 95% CI 0.43–1.60; *p* < 0.001). A significant subgroup interaction was observed for etiology (*p* < 0.001), with significant improvements in patients with traumatic brain injury and cerebrovascular accident, but not in hypoxic–ischemic brain injury. Other analyses suggested trends toward improvement in minimally conscious state, left dorsolateral prefrontal cortex stimulation, and higher total stimulation dose, without significant between-subgroup differences. No definite tDCS-related serious adverse events were reported.

**Conclusion:**

tDCS may improve the level of consciousness in patients with DoC, with no definite tDCS-related serious adverse events identified. Treatment response may differ by etiology, but this finding requires confirmation because multiple subgroup comparisons were performed.

**Systematic review registration:**

Identifier CRD420261370518.

## 1 Introduction

Disorders of consciousness (DoC) are among the most challenging conditions following severe acquired brain injury. They include coma, vegetative state/unresponsive wakefulness syndrome (VS/UWS), and minimally conscious state (MCS) (Kondziella et al., 2020). These clinical states differ in residual awareness and prognosis and may also be associated with different treatment responses (Giacino et al., 2018; Kondziella et al., 2020). Evaluating treatment effects in DoC relies largely on standardized behavioral scales because fluctuations in arousal, limited motor output, and inconsistent behavioral responses may confound clinical assessment. The Coma Recovery Scale–Revised (CRS-R) is one of the most widely used instruments for this purpose and is sensitive to changes in the level of consciousness. It is therefore commonly used as a core outcome measure in interventional DoC studies (Giacino et al., 2004).

Despite advances in the diagnosis and assessment of DoC, effective treatments to promote recovery of consciousness remain limited. Current guidelines primarily recommend amantadine for patients with traumatic VS/UWS or MCS within a specific post-injury time window. This recommendation primarily applies to subacute traumatic brain injury, whereas evidence remains insufficient for non-traumatic, chronic, or prolonged DoC (Giacino et al., 2018; Thibaut et al., 2019). The pathophysiological understanding of DoC increasingly emphasizes functional disconnection within large-scale brain networks, rather than focal structural injury alone. According to the forebrain mesocircuit model, severe brain injury may reduce cortical network activity through widespread deafferentation, reduced thalamocortical drive, and hypoactivation of prefrontal–striatal–pallidal–thalamic circuits (Edlow et al., 2021; Schiff, 2023). This framework suggests that recovery of consciousness may depend not only on the extent of structural injury but also on the capacity of residual networks to be reactivated and reintegrated. Non-invasive neuromodulation targeting key cortical nodes may therefore have therapeutic potential.

Transcranial direct current stimulation (tDCS) is a non-invasive brain stimulation technique that delivers low-intensity direct current via scalp electrodes. It can modulate cortical excitability and neuroplastic processes (Kenney-Jung et al., 2019). In studies of DoC, the dorsolateral prefrontal cortex (DLPFC) is a commonly used stimulation target, with the left DLPFC being used most frequently. The rationale for this target is supported by the involvement of the DLPFC in attention, executive control, frontoparietal networks, and thalamocortical circuits. Anodal stimulation of this region may enhance excitability within residual consciousness-related networks by modulating local cortical excitability and functional connectivity with other brain regions involved in consciousness (Kenney-Jung et al., 2019; Edlow et al., 2021). Early sham-controlled randomized studies reported that anodal tDCS over the left DLPFC could induce short-term improvement in CRS-R scores in some patients with DoC, with responses more often observed in patients with MCS (Thibaut et al., 2014). Subsequent repeated-stimulation studies suggested that several consecutive days of tDCS could elicit clinical responses in some patients with chronic MCS, and that short-term effects could be maintained after stimulation ended (Thibaut et al., 2017). More recent multicenter studies, however, did not confirm a consistent benefit at the overall group level. In contrast, studies of longer courses of repeated stimulation suggested that patients with subacute DoC may benefit. These findings suggest that treatment effects may be heterogeneous and may be influenced by baseline level of consciousness, etiology, disease duration, and stimulation protocol (Thibaut et al., 2023; Cheng et al., 2024).

Previous systematic reviews and meta-analyses have suggested that tDCS may improve the level of consciousness in patients with DoC, but their conclusions have been inconsistent (Liu et al., 2022; Fan et al., 2023; Ma et al., 2023). Some reviews reported improvements in CRS-R scores after tDCS, with potential benefits appearing more evident in patients with MCS and traumatic brain injury (TBI). Some analyses also suggested a possible benefit in patients with cerebrovascular accident (CVA) (Liu et al., 2022; Fan et al., 2023; Ma et al., 2023). A more recent updated review reached a more conservative conclusion regarding the efficacy of tDCS after restricting the analysis to sham-controlled trials, excluding studies with a high risk of bias, and addressing carry-over effects in crossover trials (Gao et al., 2025). In addition, new sham-controlled randomized trials have been published in recent years, and the existing evidence base therefore requires updating. Discrepancies across previous findings may be related to differences in comparator type, the handling of crossover trials, and the choice of outcome measures.

This study therefore systematically evaluated the effect of active tDCS versus sham tDCS on change in CRS-R score in patients with DoC, based on evidence from randomized controlled trials (RCTs). To reduce potential bias, we included only sham-controlled randomized trials for which CRS-R change scores could be extracted or calculated. For randomized crossover trials, only first-period data were extracted to minimize the influence of carry-over effects. We also conducted prespecified subgroup analyses according to baseline level of consciousness, etiology, disease duration, stimulation target, and total stimulation dose to explore potential effect modifiers of response to tDCS.

## 2 Methods

This systematic review and meta-analysis is reported in accordance with the PRISMA 2020 statement (Page et al., 2021). The protocol was registered in PROSPERO (CRD420261370518). The completed PRISMA 2020 checklist is provided in Appendix E1.

### 2.1 Search strategy

PubMed, Embase, Scopus, Web of Science, and the Cochrane Library were searched from inception to 28 May 2026. The search strategy combined terms related to tDCS, DoC, and RCTs, adapted to the syntax of each database. The full search strategies for all databases are provided in Appendix E2.

### 2.2 Eligibility criteria

Studies were eligible if they met all of the following criteria: (1) participants had clinically diagnosed DoC, including coma, VS/UWS, MCS, or other conditions consistent with the definition of DoC; (2) the experimental group received active tDCS and the control group received sham tDCS, with eligibility retained when both groups received the same background treatment or conventional rehabilitation; (3) the study design was an RCT, including randomized parallel-group trials and randomized crossover trials; (4) CRS-R data were reported, and the mean, standard deviation, and sample size for the change in CRS-R score could be extracted or calculated; and (5) the study was a peer-reviewed, full-text original article published in English.

Studies were excluded if they met any of the following criteria: (1) conference abstracts, editorials, study protocols, expert consensus statements, reviews, meta-analyses, or non-randomized controlled studies; (2) participants did not meet the definition of DoC, or data for patients with DoC could not be extracted separately; (3) the control group did not receive sham tDCS; (4) there were between-group imbalances in active neuromodulatory, pharmacological, or rehabilitation interventions other than tDCS, such that the effect of tDCS could not be isolated; (5) the change in CRS-R score was not reported and could not be calculated from original data, figures, tables, supplementary materials, or information provided by the authors; or (6) duplicate publications or overlapping samples were identified. For studies with overlapping samples, the article with the most complete data, the largest sample size, or the greatest relevance to the outcomes of this review was preferentially included.

### 2.3 Study selection and data extraction

Two reviewers independently conducted study selection and data extraction. Records were first screened by title and abstract. Full texts were then reviewed to determine eligibility against the prespecified criteria. Disagreements were resolved through discussion. If consensus could not be reached, a third reviewer adjudicated. Reasons for exclusion at the full-text stage were recorded, and the selection process is presented in a PRISMA flow diagram.

A prespecified data extraction form was used to collect study characteristics, patient characteristics, tDCS parameters, outcome data, and safety information. For trials that included tDCS, sham stimulation, and another active stimulation arm, only the tDCS-versus-sham comparison was extracted for quantitative synthesis. The primary outcome was the change in CRS-R score, defined as the post-intervention CRS-R score minus the baseline score. A positive value indicated improvement in the level of consciousness. When several CRS-R time points were reported, the change score at the end of the intervention or at the prespecified primary endpoint of the original study was preferentially extracted. If the mean and SD of the change score were reported, these values were extracted directly. If individual patient data were available, change scores were calculated for each patient and summarized as group-level mean values and SDs. If only baseline and post-intervention group-level data were available, the mean change score was calculated, and the SD of the change score was estimated using prespecified methods (Follmann et al., 1992; Pearson and Smart, 2018; Cumpston et al., 2019). Details of data conversion are provided in Appendix E3. For randomized crossover trials, only first-period data were extracted to reduce the potential influence of carry-over effects (Elbourne et al., 2002). If first-period data were unavailable, the study was excluded from the main analysis. Extracted data were cross-checked by two reviewers. Disagreements were resolved through discussion and, when necessary, adjudicated by a third reviewer.

### 2.4 Risk of bias and certainty of evidence

Risk of bias for the primary outcome in the included studies was assessed using the Cochrane Risk of Bias 2 (RoB 2) tool. The domains assessed included the randomization process, deviations from intended interventions, missing outcome data, measurement of the outcome, and selection of the reported result (Sterne et al., 2019). Each domain and the overall risk of bias were judged as low risk of bias, some concerns, or high risk of bias. For randomized crossover trials, only first-period data were included in this review. Therefore, the RoB 2 framework for parallel-group randomized trials was used to assess risk of bias for the first-period comparison. Two reviewers independently completed the risk-of-bias assessment. Disagreements were resolved through discussion and, if consensus could not be reached, adjudicated by a third reviewer. The results are presented as a risk-of-bias graph and a summary plot.

The certainty of evidence for the primary efficacy and safety outcomes was assessed using the Grading of Recommendations Assessment, Development and Evaluation (GRADE) approach (Guyatt et al., 2008). For the change in CRS-R score, judgments were based on the pooled effect estimate and the domains of risk of bias, inconsistency, indirectness, imprecision, and publication bias. For adverse events (AEs), the certainty of evidence was assessed on the basis of narrative synthesis, because reporting methods varied across studies, the number of events was small, and quantitative synthesis was not performed.

### 2.5 Statistical analysis

All analyses were performed using Stata version 16.0. For continuous outcomes, effect sizes were expressed as mean differences (MDs) with 95% confidence intervals (CIs). In multi-arm trials with more than one eligible active tDCS group sharing a single sham group, the active tDCS groups were combined into one active tDCS group according to Cochrane recommendations before comparison with the sham group. For the stimulation-target subgroup analysis, when active arms from a multi-arm trial belonged to different target categories, the shared sham group was allowed to contribute to only one target category to avoid double-counting of control participants. Statistical heterogeneity was assessed using Cochran’s Q test and the I^2^ statistic. A *p* value <0.10 for the heterogeneity test was considered to indicate statistical heterogeneity (Higgins et al., 2003). Model selection followed prespecified criteria. A fixed-effect model was used when I^2^ was <50% and the *p* value for the heterogeneity test was ≥0.10. A random-effects model was used when I^2^ was ≥50% or the *p* value for the heterogeneity test was <0.10. All statistical tests were two-sided, and *p* < 0.05 was considered statistically significant.

Subgroup analyses were conducted to explore potential effect modifiers based on baseline level of consciousness, etiology, disease duration, stimulation target, and total stimulation dose. Baseline level of consciousness was categorized as MCS or UWS/VS. Etiology was categorized as TBI, CVA, or hypoxic–ischemic brain injury (HIBI). Disease duration was categorized as ≤3 months or >3 months to distinguish patients treated in an earlier post-injury phase from those with more prolonged DoC, while maintaining sufficient data within each subgroup. Stimulation target was categorized as left DLPFC or non-left DLPFC. An operationally defined total stimulation dose was calculated as current intensity × session duration × number of sessions and was categorized as ≥800 mA·min or <800 mA·min. The cutoff of 800 mA·min corresponded to a commonly used repeated-stimulation protocol of 2 mA for 20 min over 20 sessions. Subgroup analyses preferentially used patient-level stratified efficacy data reported in the original studies. When stratified efficacy data were unavailable but the overall study population could be clearly assigned to a subgroup, classification was based on study-level characteristics. Studies that could not be clearly classified were excluded from the corresponding subgroup analysis. Between-subgroup differences were assessed using tests of subgroup differences.

Sensitivity analyses were performed using a leave-one-out method. Additional sensitivity analyses were conducted by excluding single-session studies, very small studies or comparisons, and studies requiring estimation of the SD of the change score. When more than 10 studies were included in the main analysis, publication bias and small-study effects were assessed using a funnel plot and Egger’s test. When appropriate, the trim-and-fill method was used as an exploratory sensitivity analysis (Egger et al., 1997; Duval and Tweedie, 2000). Because adverse event reporting was inconsistent across studies and the number of events per group was insufficient, adverse events were not quantitatively pooled and were summarized descriptively.

## 3 Results

### 3.1 Study selection

**Figure 1:**
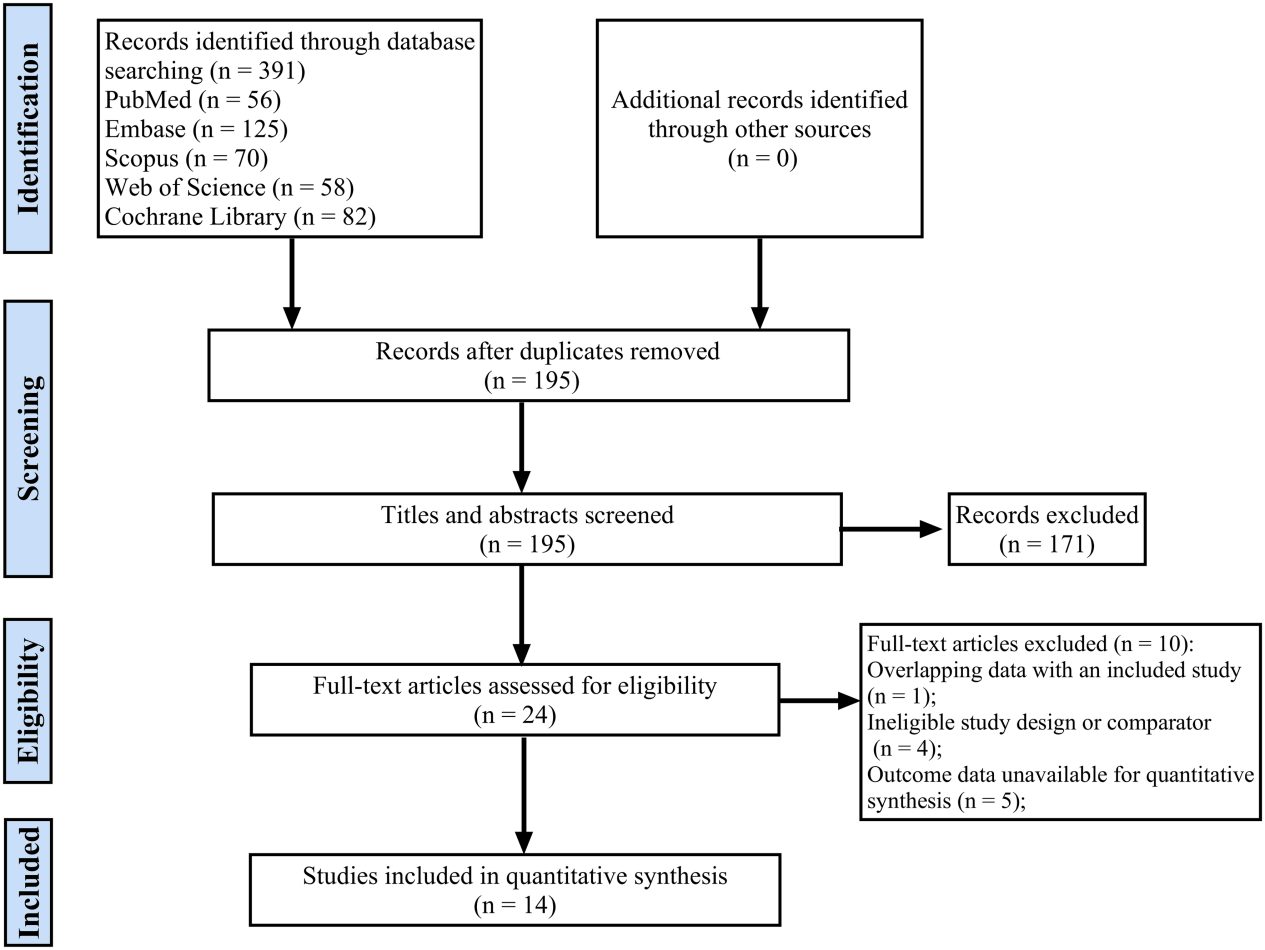
PRISMA 2020 flow diagram of study selection. The flow diagram shows database searching, deduplication, screening, full-text assessment, and inclusion in the quantitative synthesis.

The database search identified 391 records: 56 from PubMed, 125 from Embase, 70 from Scopus, 58 from Web of Science, and 82 from the Cochrane Library. No additional records were identified from other sources. After all records were imported into EndNote 21 and duplicates were removed, 195 records remained. Title and abstract screening excluded 171 records that did not meet the eligibility criteria, leaving 24 articles for full-text assessment. After full-text review, 10 articles were excluded: one because of overlapping data with an included study, four because the study design or comparator did not meet the eligibility criteria, and five because outcome data suitable for meta-analysis could not be extracted. Overall, 14 studies met the eligibility criteria and were included in the quantitative synthesis (Figure 1).

### 3.2 Characteristics of included studies

Fourteen sham-controlled RCTs were included, enrolling 330 patients with DoC. Of these, 173 patients were assigned to active tDCS and 157 to sham tDCS. Sample sizes ranged from 7 to 52 participants per study. Six studies used a parallel-group design, and eight used a randomized crossover design. The main characteristics of the included studies are shown in Table 1. Detailed study characteristics are provided in Appendix E4 (Table E4.1).

**Table 1:** Characteristics of included sham-controlled randomized trials.

Study	Design	Sample size (*n*)	Sex (M/F)	Age (years)	DoC type	Disease duration (months)	Etiology
Zhang et al. (2017)	Parallel RCT	G1: 13	7/6	58.54 ± 15.78	MCS 8; VS 5	5.70 ± 4.60	Hypoxia 2; TBI 5; hemorrhage 5; ischemia 1
		G2: 13	8/5	46.85 ± 22.90	MCS 7; VS 6	5.00 ± 3.80	Hypoxia 3; TBI 7; hemorrhage 2; ischemia 1
Yu et al. (2022)	Parallel RCT	G1: 15	15/0	51.73 ± 9.40	VS 15	6.35 ± 5.42	Stroke 15
		G2: 16	13/3	59.38 ± 9.50	VS 16	6.00 ± 3.21	Stroke 16
Wu et al. (2019)	Parallel RCT	G1A: 5	4/1	44.40 ± 27.25	MCS 3; UWS 2	152.40 ± 98.71	Hemorrhage 3; trauma 2
		G1B: 5	2/3	54.80 ± 12.68	MCS 1; UWS 4	227.80 ± 234.75	Hemorrhage 4; trauma 1
		G2: 5	3/2	44.40 ± 10.95	MCS 3; UWS 2	78.20 ± 58.42	Anoxia 3; trauma 2
Thibaut et al. (2017)	Crossover RCT	G1: 9	5/4	40.89 ± 11.24	MCS 9	94.00 ± 113.37	TBI 6; cardiac arrest 2; anoxic stroke 1
		G2: 7	4/3	46.43 ± 20.58	MCS 7	58.00 ± 86.18	TBI 5; cardiac arrest 1; anoxic stroke 1
Thibaut et al. (2023)	Parallel RCT	G1: 29	21/8	41.10 ± 15.33	MCS 13; UWS/VS 16	10.09 ± 6.71	TBI 12; anoxic/hypoxic 14; CVA/vascular 3
		G2: 23	14/9	44.61 ± 13.41	MCS 11; UWS/VS 12	7.11 ± 5.19	TBI 7; anoxic/hypoxic 9; CVA/vascular 7
Martens et al. (2018)	Crossover RCT	G1: 16	10/6	47.56 ± 15.71	MCS 16	79.00 ± 63.18	TBI 3; ischemic stroke 2; cardiac arrest 7; aneurysm 3; anoxia 1
		G2: 11	9/2	33.91 ± 7.25	MCS 11	122.91 ± 103.75	TBI 9; cardiac arrest 2
Martens et al. (2020)	Crossover RCT	G1: 24	15/9	48.75 ± 13.63	MCS 16; UWS/VS 8	53.71 ± 83.98	TBI 12; non-TBI 12
		G2: 16	9/7	43.81 ± 16.31	MCS 7; UWS/VS 9	14.86 ± 17.36	TBI 4; non-TBI 12
Martens et al. (2019)	Crossover RCT	G1: 4	2/2	53.25 ± 21.28	MCS 2; UWS/VS 2	3.11 ± 4.21	TBI 2; non-TBI 2
		G2: 6	6/0	46.33 ± 25.07	MCS 4; UWS/VS 2	9.84 ± 16.81	TBI 3; non-TBI 3
Huang et al. (2017)	Crossover RCT	G1: 15	10/5	54.33 ± 13.24	MCS 15	6.60 ± 5.82	Trauma 7; CVA 6; subarachnoid hemorrhage 1; hypoxic–ischemic 1
		G2: 18	10/8	59.11 ± 8.41	MCS 18	4.94 ± 3.64	Trauma 13; CVA 4; hypoxic–ischemic 1
Estraneo et al. (2017)	Crossover RCT	G1: 8	6/2	54.13 ± 23.79	VS 5; MCS 3	16.88 ± 27.47	Traumatic 1; vascular 3; anoxic 4
		G2: 5	1/4	55.20 ± 20.32	VS 2; MCS 3	25.00 ± 20.86	Anoxic 2; vascular 3
Cheng et al. (2024)	Parallel RCT	G1: 14	11/3	44.21 ± 15.49	MCS 5; UWS/VS 9	1.73 ± 0.69	TBI 7; hemorrhagic 6; anoxia 1
		G2: 14	11/3	46.71 ± 14.33	MCS 4; UWS/VS 10	2.00 ± 1.15	TBI 8; hemorrhagic 3; anoxia 3
Carrière et al. (2020)	Crossover RCT	G1: 3	3/0	45.67 ± 15.31	MCS 3	11.67 ± 11.55	TBI 2; aneurysm 1
		G2: 7	4/3	47.57 ± 16.25	MCS 7	6.71 ± 4.42	Cardiac arrest 2; aneurysm 3; meningitis 1; TBI 1
Barra et al. (2022)	Crossover RCT	G1: 2	0/2	41.00 ± 14.14	MCS 2	4.50 ± 0.71	Stroke 1; anoxic 1
		G2: 5	2/3	51.40 ± 18.35	MCS 5	4.66 ± 4.57	Stroke 3; TBI 2
Ma et al. (2026)	Parallel RCT	G1: 11	8/3	49.45 ± 13.90	NR	0.47 ± 0.24	Traumatic 6; vascular 4; other 1
		G2: 11	5/6	47.27 ± 16.06	NR	0.49 ± 0.20	Traumatic 1; vascular 9; other 1

G1 and G2 indicate the active and sham tDCS groups, respectively; in Wu et al. (2019), G1A and G1B indicate the left- and right-DLPFC active groups. Etiology is reported as described in the original studies. CVA, cerebrovascular accident; DLPFC, dorsolateral prefrontal cortex; DoC, disorders of consciousness; HIBI, hypoxic–ischemic brain injury; MCS, minimally conscious state; NR, not reported; RCT, randomized controlled trial; TBI, traumatic brain injury; UWS/VS, unresponsive wakefulness syndrome/vegetative state.

Participants were mainly patients with MCS or VS/UWS. Etiologies included TBI, CVA, HIBI, and other acquired brain injuries. Disease duration covered acute, subacute, and chronic stages. The most common stimulation target was the left DLPFC. Other targets included the right DLPFC, primary motor cortex, posterior parietal cortex, left prefrontal cortex, and bilateral frontoparietal networks. Most studies used a stimulation protocol of 2 mA for 20 min per session. Four studies used single-session stimulation, whereas the remaining studies used repeated stimulation; across all studies, the total number of sessions ranged from 1 to 20. Detailed stimulation parameters are provided in Appendix E4 (Table E4.2).

### 3.3 Risk of bias assessment

**Figure 2:**
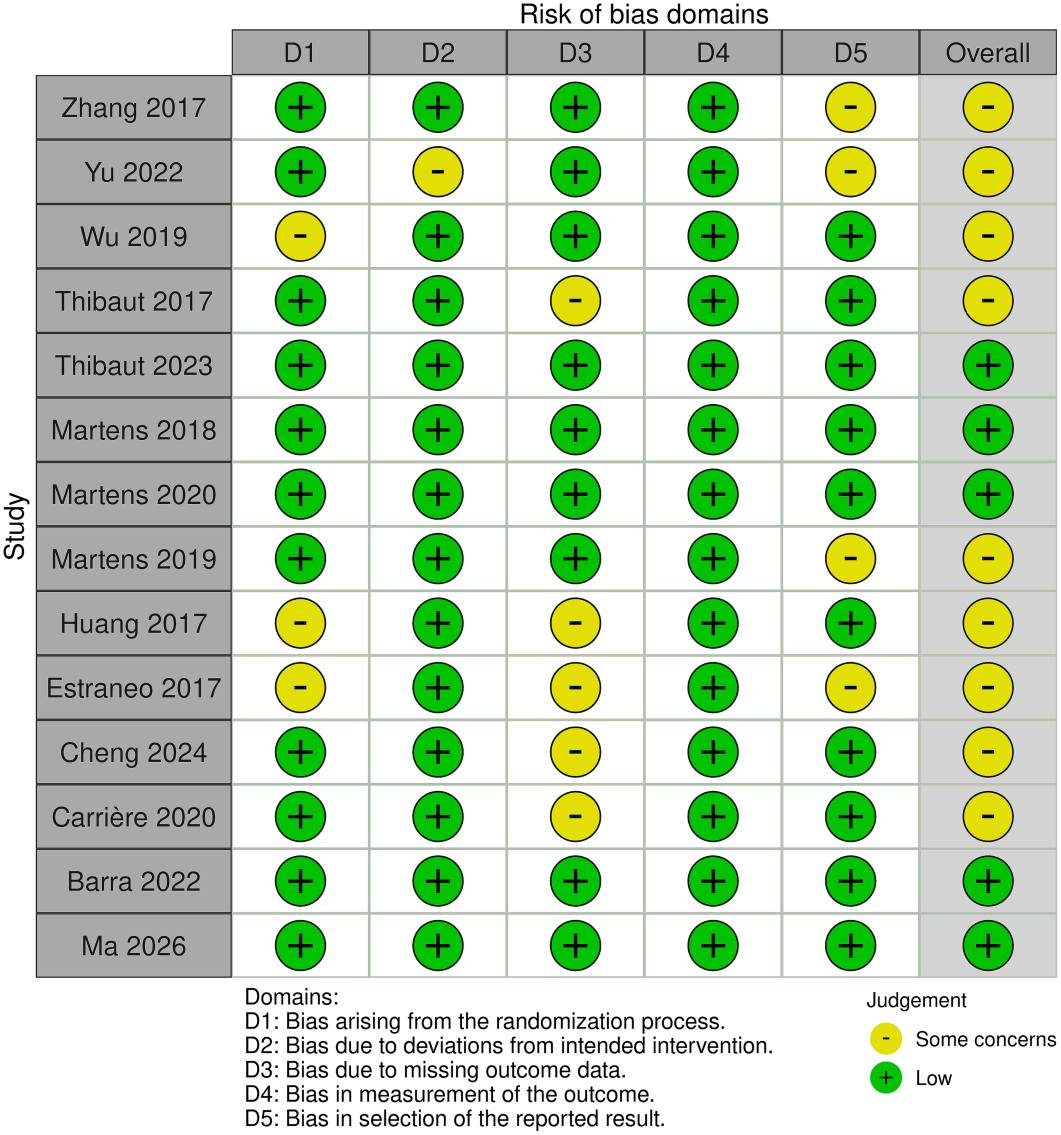
Risk-of-bias judgments for the primary outcome in individual studies. Risk of bias was assessed using the Cochrane RoB 2 tool for Coma Recovery Scale–Revised change scores.

**Figure 3:**
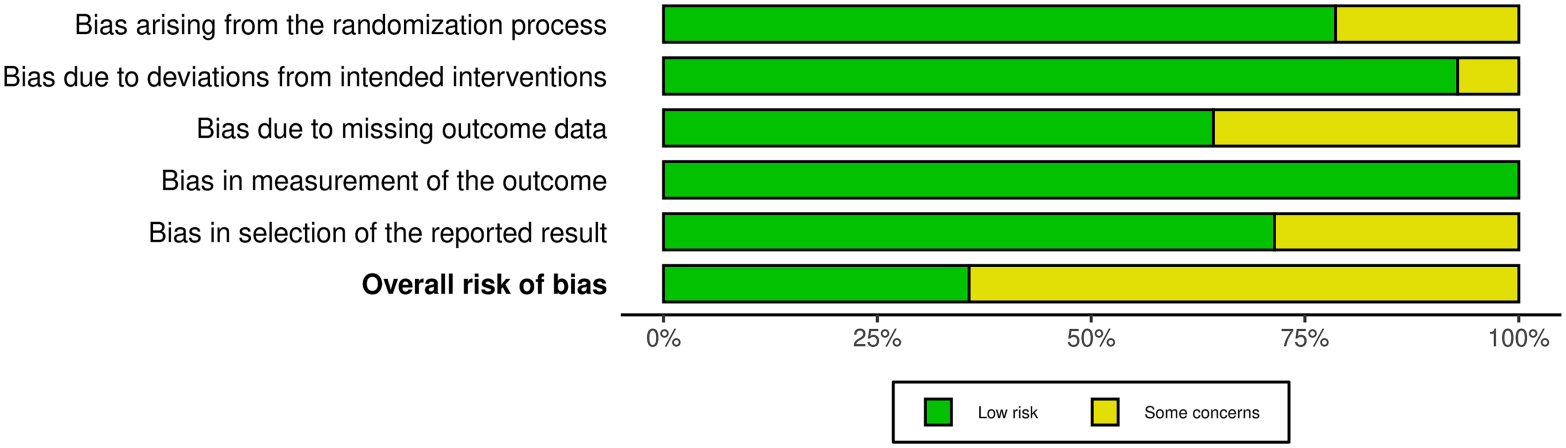
Summary of risk-of-bias judgments for the primary outcome. The figure shows the proportions of studies rated as low risk, some concerns, or high risk across RoB 2 domains and overall.

Risk of bias was assessed for the 14 studies using the Cochrane RoB 2 tool. Five studies were judged to have a low risk of bias (Martens et al., 2018; Martens et al., 2020; Barra et al., 2022; Thibaut et al., 2023; Ma et al., 2026), and nine were judged to have some concerns (Estraneo et al., 2017; Huang et al., 2017; Thibaut et al., 2017; Zhang et al., 2017; Martens et al., 2019; Wu et al., 2019; Carrière et al., 2020; Yu et al., 2022; Cheng et al., 2024). No study was judged to have a high risk of bias. The main concerns related to insufficient reporting of the randomization process, missing outcome data, and the absence of a registered protocol or prespecified statistical analysis plan. All studies were judged to have a low risk of bias in the measurement-of-the-outcome domain. Figure 2 shows the domain-level risk-of-bias judgments for each study. Figure 3 shows the overall distribution of risk of bias. Supporting reasons are provided in Appendix E5.

### 3.4 Effect of tDCS on change in CRS-R score

**Figure 4:**
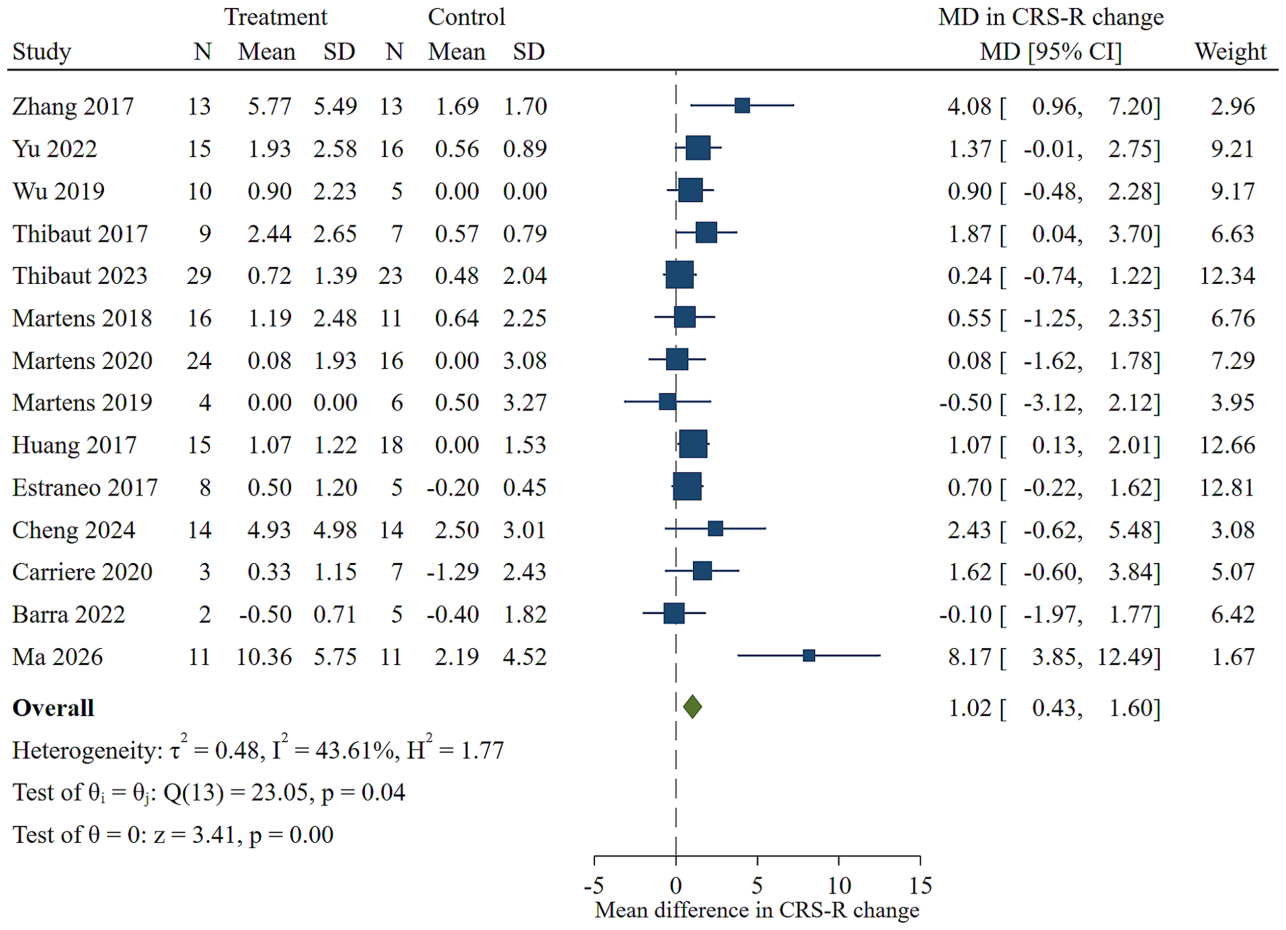
Forest plot of active versus sham tDCS for CRS-R change scores. Effect sizes are expressed as MDs with 95% CIs. Values greater than zero favor active stimulation.

Fourteen studies (Estraneo et al., 2017; Huang et al., 2017; Thibaut et al., 2017; Zhang et al., 2017; Martens et al., 2018; Martens et al., 2019; Wu et al., 2019; Carrière et al., 2020; Martens et al., 2020; Barra et al., 2022; Yu et al., 2022; Thibaut et al., 2023; Cheng et al., 2024; Ma et al., 2026) were included in the quantitative synthesis of the change in CRS-R score, comprising 173 patients in the active tDCS group and 157 in the sham tDCS group. The heterogeneity test indicated moderate between-study heterogeneity (I^2^ = 43.61%, *p* = 0.041); therefore, a random-effects model was used. Compared with sham tDCS, active tDCS was associated with a significantly greater improvement in the change in CRS-R score (MD = 1.02, 95% CI 0.43–1.60; *p* < 0.001) (Figure 4).

### 3.5 Subgroup analyses

**Figure 5:**
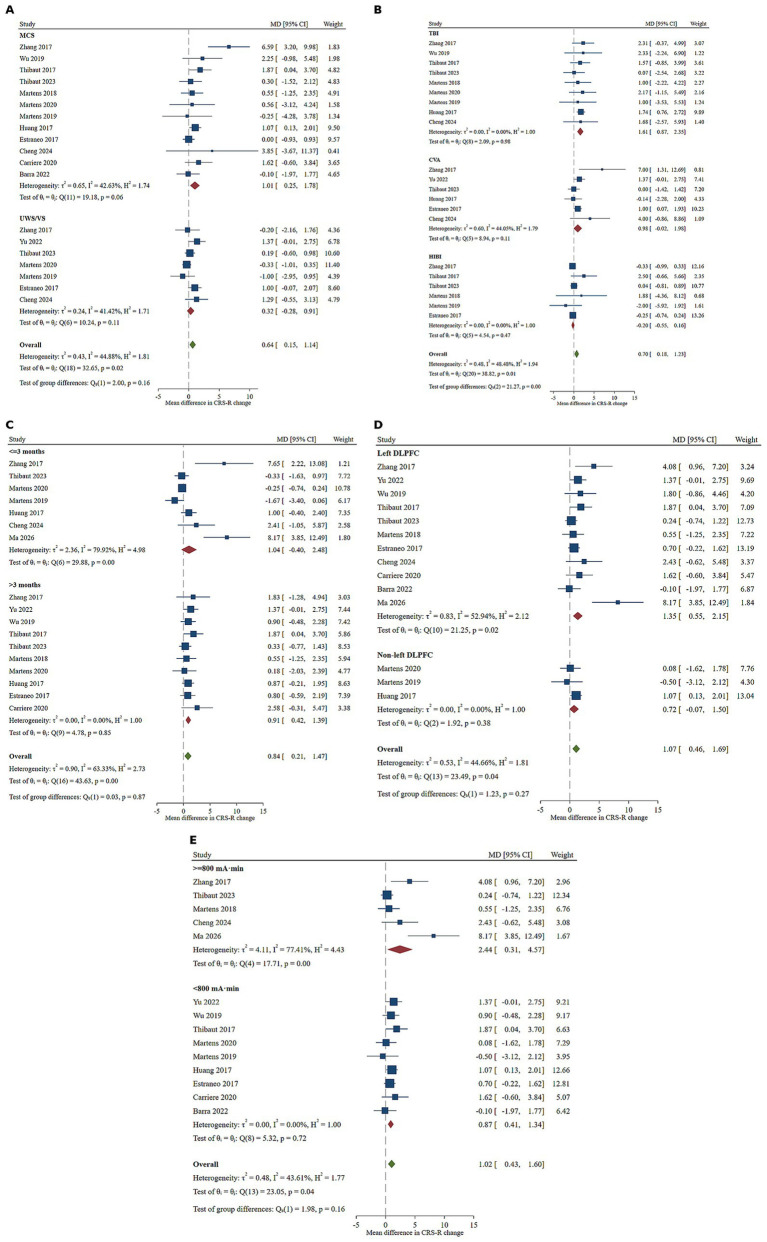
Subgroup forest plots for CRS-R change scores. **(A)** Baseline level of consciousness; **(B)** etiology; **(C)** disease duration; **(D)** stimulation target; **(E)** total stimulation dose.

Results of the subgroup analyses are shown in Table 2 and Figure 5. The individual subgroup forest plots are provided separately in Appendix E6. When stratified by baseline level of consciousness, the MCS subgroup included 12 comparisons (Estraneo et al., 2017; Huang et al., 2017; Thibaut et al., 2017; Zhang et al., 2017; Martens et al., 2018; Martens et al., 2019; Wu et al., 2019; Carrière et al., 2020; Martens et al., 2020; Barra et al., 2022; Thibaut et al., 2023; Cheng et al., 2024). Active tDCS was associated with a significantly greater improvement in the change in CRS-R score than sham tDCS (MD = 1.01, 95% CI 0.25–1.78). The UWS/VS subgroup included seven comparisons (Estraneo et al., 2017; Zhang et al., 2017; Martens et al., 2019; Martens et al., 2020; Yu et al., 2022; Thibaut et al., 2023; Cheng et al., 2024), with no statistically significant benefit observed (MD = 0.32, 95% CI − 0.28–0.91). The between-subgroup difference was not statistically significant (*p* = 0.16).

**Table 2:** Subgroup analyses based on CRS-R change scores.

Factor	Subgroup	No.	MD (95% CI)	I^2^	P for interaction
Consciousness level	MCS	12	1.01 (0.25–1.78)	42.63%	0.16
	UWS/VS	7	0.32 (−0.28–0.91)	41.42%	
Etiology	TBI	9	1.61 (0.87–2.35)	0.00%	<0.001
	CVA	6	0.90 (0.27–1.54)	44.05%	
	HIBI	6	−0.20 (−0.55–0.16)	0.00%	
Disease duration	≤3 months	7	1.04 (−0.40–2.48)	79.92%	0.87
	>3 months	10	0.91 (0.42–1.39)	0.00%	
Stimulation target	Left DLPFC	11	1.35 (0.55–2.15)	52.94%	0.27
	Non-left DLPFC	3	0.72 (−0.07–1.50)	0.00%	
Total stimulation dose	≥800 mA·min	5	2.44 (0.31–4.57)	77.41%	0.16
	<800 mA·min	9	0.87 (0.41–1.34)	0.00%	

MD >0 indicates greater improvement in CRS-R change scores in the active tDCS group than in the sham group. No. indicates the number of study-level or subgroup-level comparisons included in each subgroup.

When stratified by etiology, the TBI subgroup included nine comparisons (Huang et al., 2017; Thibaut et al., 2017; Zhang et al., 2017; Martens et al., 2018; Martens et al., 2019; Wu et al., 2019; Martens et al., 2020; Thibaut et al., 2023; Cheng et al., 2024) and showed a significant improvement in the change in CRS-R score (MD = 1.61, 95% CI 0.87–2.35). The CVA subgroup included six comparisons (Estraneo et al., 2017; Huang et al., 2017; Zhang et al., 2017; Yu et al., 2022; Thibaut et al., 2023; Cheng et al., 2024) and also showed a significant improvement (MD = 0.90, 95% CI 0.27–1.54). The HIBI subgroup included six comparisons (Estraneo et al., 2017; Thibaut et al., 2017; Zhang et al., 2017; Martens et al., 2018; Martens et al., 2019; Thibaut et al., 2023), with no statistically significant benefit observed (MD = −0.20, 95% CI − 0.55–0.16; *p* = 0.27). The between-subgroup difference by etiology was statistically significant (*p* < 0.001).

When stratified by disease duration, the ≤3-month subgroup included seven comparisons (Huang et al., 2017; Zhang et al., 2017; Martens et al., 2019; Martens et al., 2020; Thibaut et al., 2023; Cheng et al., 2024; Ma et al., 2026). No statistically significant benefit was observed, and heterogeneity was high (MD = 1.04, 95% CI − 0.40–2.48). The >3-month subgroup included 10 comparisons (Estraneo et al., 2017; Huang et al., 2017; Thibaut et al., 2017; Zhang et al., 2017; Martens et al., 2018; Wu et al., 2019; Carrière et al., 2020; Martens et al., 2020; Yu et al., 2022; Thibaut et al., 2023) and showed a statistically significant benefit (MD = 0.91, 95% CI 0.42–1.39). The between-subgroup difference was not statistically significant (*p* = 0.87).

When stratified by stimulation target, the left DLPFC subgroup included 11 comparisons (Estraneo et al., 2017; Thibaut et al., 2017; Zhang et al., 2017; Martens et al., 2018; Wu et al., 2019; Carrière et al., 2020; Barra et al., 2022; Yu et al., 2022; Thibaut et al., 2023; Cheng et al., 2024; Ma et al., 2026) and showed a statistically significant benefit (MD = 1.35, 95% CI 0.55–2.15). The non-left DLPFC subgroup included three comparisons (Huang et al., 2017; Martens et al., 2019; Martens et al., 2020), and the effect did not reach statistical significance (MD = 0.72, 95% CI − 0.07–1.50). The between-subgroup difference was not statistically significant (*p* = 0.27).

When stratified by total stimulation dose, the ≥ 800 mA·min subgroup included five comparisons (Zhang et al., 2017; Martens et al., 2018; Thibaut et al., 2023; Cheng et al., 2024; Ma et al., 2026) and showed a larger effect estimate with high heterogeneity (MD = 2.44, 95% CI 0.31–4.57). The < 800 mA·min subgroup included nine comparisons (Estraneo et al., 2017; Huang et al., 2017; Thibaut et al., 2017; Martens et al., 2019; Wu et al., 2019; Carrière et al., 2020; Martens et al., 2020; Barra et al., 2022; Yu et al., 2022) and also showed a statistically significant benefit (MD = 0.87, 95% CI 0.41–1.34). The between-subgroup difference did not reach statistical significance (*p* = 0.16).

### 3.6 Sensitivity analyses

The sensitivity analysis results are provided in Appendix E7. In the leave-one-out analysis, pooled effect estimates ranged from MD = 0.83 to MD = 1.14, and all estimates remained statistically significant. After excluding single-session studies, very small studies or comparisons, or studies requiring estimation of the SD of the change score, the pooled results remained consistent with the main analysis. Excluding Ma et al. (2026) markedly reduced between-study heterogeneity (I^2^ = 0.53%), suggesting that this study may have been a major source of heterogeneity. Overall, these analyses supported the robustness of the main findings.

### 3.7 Publication bias and small-study effects

**Figure 6:**
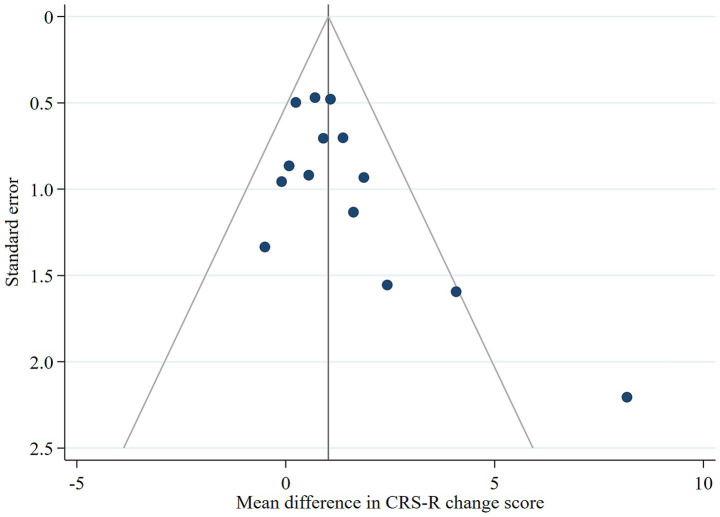
Funnel plot for the primary analysis of CRS-R change scores. The funnel plot was used to assess potential publication bias or small-study effects.

For the primary outcome, the funnel plot showed some asymmetry in the distribution of studies (Figure 6). Egger’s regression test suggested small-study effects (*p* = 0.0095), whereas Begg’s rank correlation test did not reach statistical significance (*p* = 0.0798) (Appendix E8). The trim-and-fill method was then used as an exploratory sensitivity analysis. This analysis estimated that two studies may have been missing on the left side of the funnel plot. After adjustment, the pooled effect estimate decreased from MD = 1.018 (95% CI 0.433–1.604) to MD = 0.806 (95% CI 0.091–1.521) (Appendix E8). After trim-and-fill adjustment, the pooled effect estimate was attenuated, but the direction of effect remained unchanged and the adjusted estimate remained statistically significant.

### 3.8 Safety and adverse events

AEs were summarized descriptively because definitions, recording methods, and group-specific reporting varied across trials, and several crossover trials did not report first-period safety data separately. Among the 14 included trials, 11 provided some information on AEs, whereas three did not provide extractable group-specific safety data. Seven trials reported no AEs or no tDCS-related adverse reactions. Four trials reported mild and transient adverse reactions, including skin redness, tingling, itching, paresthesia, somnolence, or discomfort at the electrode site. No definite tDCS-related serious adverse events were reported.

### 3.9 GRADE certainty of evidence

The GRADE assessment is provided in Appendix E9. The certainty of evidence for the change in CRS-R score was low after downgrading one level for risk of bias and one level for possible publication bias or small-study effects. The certainty of evidence for AEs was very low. The main reasons were insufficient reporting of safety outcomes, inconsistent recording and group-specific AE reporting across studies, a small number of events, and the absence of quantitative synthesis.

## 4 Discussion

### 4.1 Main findings

This systematic review and meta-analysis included 14 sham-controlled randomized trials enrolling 330 patients with DoC. Compared with sham tDCS, active tDCS was associated with greater improvement in the change in CRS-R score. Subgroup analyses showed trends toward improvement in the MCS, TBI, CVA, left DLPFC stimulation, and higher total stimulation dose subgroups. However, only the between-subgroup difference for etiology reached statistical significance. Regarding safety, the included studies did not report definite tDCS-related serious AEs. The safety evidence remains limited because adverse events were insufficiently monitored, recorded, and reported by treatment group.

### 4.2 Interpretation of efficacy and potential effect modifiers

The effect of tDCS in DoC may depend in part on the modifiability of residual consciousness-related networks. DoC does not represent a single pathological state. Treatment response may be influenced by etiology, lesion distribution, disease stage, and the functional state of residual networks. According to the forebrain mesocircuit model, severe brain injury may lead to widespread deafferentation, reduced thalamocortical drive, and hypoactivation of prefrontal–striatal–pallidal–thalamic circuits. These changes may leave residual cortical networks with reduced excitability and integration (Edlow et al., 2021; Schiff, 2023). In this context, tDCS may promote reactivation of consciousness-related networks with preserved structural and functional connectivity by modulating cortical excitability (Nitsche and Paulus, 2000; Edlow et al., 2021). However, the pooled estimate was derived from CRS-R change scores, which capture examiner-rated behavioral responsiveness rather than direct physiological network activity and may be affected by arousal fluctuations, motor-output limitations, and assessor judgment. Thus, within the present meta-analysis, network reactivation should be regarded as a mechanistic interpretation rather than a direct finding from the pooled CRS-R outcome. This framework may help explain why behavioral improvement has been observed only in some patients in previous studies of DLPFC-tDCS (Thibaut et al., 2014; Thibaut et al., 2017; Thibaut et al., 2023; Cheng et al., 2024).

Only a few included trials reported objective neurophysiological outcomes alongside CRS-R assessments. One trial reported CRS-R improvement in MCS patients after active tDCS, with increased P300 amplitude but no significant change in P300 latency, suggesting partial concordance between behavioral and ERP findings (Zhang et al., 2017). Another trial reported preliminary hdEEG changes after a single active tDCS session, including uncorrected increases in alpha- and theta-band power and alpha-band connectivity, but found neither corrected group-level EEG effects nor significant group-level CRS-R treatment effects (Carrière et al., 2020). Taken together, these findings provide limited and heterogeneous instrument-based support for network-level modulation. They also suggest that neurophysiological changes may not consistently parallel examiner-rated behavioral changes.

In the main analysis, the pooled MD for the change in CRS-R score was approximately 1 point. The clinical interpretation of this effect size should consider the structure and scoring properties of the CRS-R. The CRS-R comprises six subscales assessing auditory, visual, motor, oromotor/verbal, communication, and arousal functions. Items within each subscale are arranged hierarchically. Thus, a change in the total score may reflect the emergence of a key consciousness-related behavior rather than a simple linear improvement in severity (Giacino et al., 2004). In patients with DoC, a small change in CRS-R score may correspond to a new threshold-level behavior or a change in diagnostic status. Nevertheless, a group-level MD of approximately 1 point should not be directly equated with stable recovery of consciousness or improved long-term prognosis (Monti et al., 2023; Weaver et al., 2024). tDCS may therefore induce observable short-term changes in consciousness-related behaviors in some patients. Whether these changes represent clinically important improvement at the individual level should be judged in relation to CRS-R subscale changes, diagnostic transitions, responder rates, and long-term follow-up outcomes.

Baseline level of consciousness may be an important source of heterogeneity in the efficacy of tDCS. Previous RCTs and meta-analyses have generally suggested that patients with MCS are more likely to show tDCS-related behavioral improvement than patients with UWS/VS (Thibaut et al., 2014; Liu et al., 2022; Fan et al., 2023; Ma et al., 2023). Repeated-stimulation studies have also shown short-term benefit in some patients with chronic MCS (Thibaut et al., 2017). In the present study, the MCS subgroup showed statistically significant improvement, whereas the UWS/VS subgroup showed no statistically significant benefit. This pattern of effect is consistent with previous evidence. A possible explanation is that patients with MCS retain more consciousness-related networks and behavioral output pathways and may therefore have greater potential to respond to neuromodulation. However, the between-subgroup difference for MCS versus UWS/VS did not reach statistical significance. The current evidence is therefore insufficient to confirm baseline level of consciousness as a definite modifier of the tDCS response. MCS may therefore be regarded as a subgroup with greater response potential, but this finding should not be used to exclude the possibility of benefit in some patients with UWS/VS.

Etiology showed the clearest subgroup signal in this study. The TBI and CVA subgroups showed statistically significant improvement, whereas the HIBI subgroup did not show a statistically significant benefit. The between-subgroup difference by etiology was statistically significant. However, because this was the only significant interaction among several subgroup analyses conducted within a limited evidence base, the possibility of multiple-comparison risk should be considered. Therefore, the etiology finding should be interpreted cautiously rather than as confirmatory evidence of effect modification. Nevertheless, the observed pattern is biologically plausible. Although TBI and some forms of CVA may involve widespread brain networks, residual networks and the capacity for reorganization may still be preserved to some extent. In contrast, HIBI is often associated with global cerebral hypoxia, hypoperfusion, or both. In adults, global hypoxic–ischemic brain injury may involve the diffuse cortex, deep gray matter, white matter, brainstem, cerebellum, or hippocampus and is associated with poorer clinical outcomes (Muttikkal and Wintermark, 2013). These injury patterns may weaken the basis for reactivation of thalamocortical and corticocortical networks (Muttikkal and Wintermark, 2013; Edlow et al., 2021; Schiff, 2023). Differences by etiology may therefore reflect differences in network preservation, lesion distribution, and recovery potential across brain injury types. This finding suggests that future studies of tDCS efficacy should consider etiology and the underlying patterns of network injury, rather than simply pooling patients under the broad diagnosis of DoC.

Disease duration may influence the apparent treatment effect of tDCS, but the current evidence does not identify a disease stage at which patients consistently derive greater benefit. Earlier-stage DoC may retain greater recovery potential and residual network plasticity, while also being more susceptible to spontaneous neurological recovery, arousal instability, and short-term CRS-R fluctuations. In more prolonged DoC, spontaneous recovery is less prominent, although treatment response may be constrained by more established structural and network injury. One pilot RCT enrolling patients with acute DoC and mean disease durations of approximately 0.5 months in both arms was the main contributor to heterogeneity in the leave-one-out analysis (Ma et al., 2026). Therefore, the large effect observed in this trial may not be attributable to tDCS alone, but may also have been influenced by spontaneous recovery and baseline prognostic differences in acute-stage patients.

Stimulation target and total stimulation dose provide directional signals, but strong inferences are not warranted at present. The left DLPFC subgroup showed improvement, consistent with the theoretical role of prefrontal–thalamocortical networks in recovery of consciousness (Edlow et al., 2021; Schiff, 2023). However, the non-left DLPFC subgroup included few studies and heterogeneous stimulation targets. The between-subgroup difference did not reach statistical significance, and the current evidence does not show that left DLPFC stimulation is superior to other targets. Similarly, the ≥ 800 mA·min subgroup showed a larger effect estimate, but heterogeneity was high and the between-subgroup difference by dose was not statistically significant. Thus, the current findings suggest only that stimulation target and stimulation dose may influence treatment effects. They are insufficient to determine the optimal target or an effective dose threshold.

### 4.3 Comparison with previous studies

The overall direction of the present findings is broadly consistent with previous systematic reviews on tDCS in DoC. Earlier meta-analyses generally suggested that tDCS may improve the level of consciousness in patients with DoC, with benefits more likely to be observed in patients with MCS and TBI. Some analyses also suggested a possible benefit in patients with CVA (Liu et al., 2022; Fan et al., 2023; Ma et al., 2023). In the present study, active tDCS still showed a positive effect after the analysis was restricted to sham-controlled trials, the change in CRS-R score was used as the outcome, and only first-period data from crossover trials were included. These findings are consistent with the possibility that tDCS may produce behavioral benefits in some patients with DoC.

Differences in conclusions across meta-analyses may be related to differences in eligibility criteria, comparator type, the handling of crossover trials, and outcome definitions (Liu et al., 2022; Fan et al., 2023; Ma et al., 2023; Gao et al., 2025). This issue is particularly relevant when comparing the present study with Ma et al. (2023), the most closely related previous meta-analysis. That review searched the literature up to June 2022, identified 17 eligible studies, and included 15 studies in the quantitative synthesis, with subgroup analyses mainly by stimulation protocol, stimulation dose or session number, and patient diagnosis. By contrast, the present review updated the search, restricted the quantitative synthesis to sham-controlled RCTs with extractable or calculable CRS-R change-score data, included only first-period data from randomized crossover trials, and explored potential effect modifiers according to baseline level of consciousness, etiology, disease duration, stimulation target, and total stimulation dose.

Therefore, the main added value of the present study lies not only in the updated search but also in the re-estimation of tDCS effects within a more consistent sham-controlled framework. A recent updated review reached a more conservative conclusion after applying stricter eligibility criteria and crossover-trial handling methods (Gao et al., 2025), suggesting that estimates of tDCS efficacy are sensitive to study inclusion, outcome definition, and the handling of crossover designs. Taken together, these differences show that the present review is not merely an expansion of the evidence base, but a methodologically more restricted update that re-estimates the effect of active tDCS using sham-controlled comparisons, a uniform CRS-R change-score outcome, and conservative handling of crossover trials.

### 4.4 Clinical implications and safety

The findings of this study suggest that tDCS may be considered a potential adjunctive neuromodulatory strategy in rehabilitation for DoC, rather than a stand-alone arousal-promoting treatment or a routine, non-selective intervention. The key issue for clinical application is not broadening its use indiscriminately but identifying patients who are more likely to benefit. Based on the present exploratory findings and previous mechanistic evidence, future confirmatory studies may prioritize patients with MCS, TBI, or CVA, as well as those with residual behavioral fluctuations, electroencephalographic reactivity, or preserved functional connectivity. For patients with diffuse HIBI or a long-standing absence of reproducible behavioral and neurophysiological responses, treatment expectations should be set cautiously.

Regarding safety, the included studies did not report definite tDCS-related serious AEs. Reported adverse reactions were mostly mild and transient, including skin redness, tingling, itching, paresthesia, and discomfort at the electrode site. These findings suggest that, within the scope of available reporting, no clear serious safety signal was observed for tDCS. However, AE definitions, active monitoring methods, assessment time points, and group-specific reporting were inconsistent across studies. In addition, patients with DoC may have limited ability to report subjective discomfort. The current evidence is therefore insufficient to fully establish the safety of tDCS in this population.

### 4.5 Strengths and limitations

This study has several methodological strengths. First, only comparisons of active tDCS with sham tDCS were included, which reduced the influence of conventional rehabilitation, spontaneous recovery, and non-specific differences in care on the effect estimates. Second, the primary outcome was consistently defined as the change in CRS-R score, which better reflects within-patient change before and after treatment than post-intervention scores alone. Third, only first-period data were extracted from randomized crossover trials to reduce potential carry-over effects. Fourth, this study included recently published sham-controlled randomized trials and assessed the evidence using the RoB 2 tool and the GRADE framework. Fifth, subgroup analyses were conducted according to level of consciousness, etiology, disease duration, stimulation target, and total stimulation dose, allowing the findings to be interpreted in relation to clinically relevant sources of heterogeneity in DoC neuromodulation.

This study also has limitations. Although more studies were included than in previous reviews, the total sample remained limited, individual trials were generally small, and subgroup analyses were likely underpowered. Because several subgroup analyses were conducted within the same small evidence base, multiple-comparison risk may reduce confidence in the etiology finding. The consciousness-level subgroup analysis also involved potential circularity because MCS versus UWS/VS classification is commonly based on CRS-R behavioral criteria, whereas CRS-R change was the primary outcome. Clinical heterogeneity was present in etiology, disease stage, background treatment, stimulation parameters, and sham protocols. The dose subgroup analysis was exploratory because total dose was defined as a simple linear composite of current intensity, session duration, and number of sessions, without accounting for electrode montage, current density, or individual electric-field distribution. AEs were insufficiently reported, precluding quantitative synthesis and limiting safety conclusions. Evidence was largely restricted to short-term CRS-R changes, with little information on long-term diagnostic transitions, functional communication, quality of life, or caregiver burden. Finally, funnel plot asymmetry and Egger’s test suggested possible publication bias or small-study effects, while the attenuation after trim-and-fill indicated that the unadjusted estimate may overstate the true efficacy of tDCS. However, these findings remain uncertain because Egger’s test was applied close to its lower recommended threshold and trim-and-fill estimates may be unstable with only 14 studies. Larger, prospectively registered, multicenter sham-controlled trials are needed to provide more reliable estimates of treatment effect.

## 5 Conclusion

This systematic review and meta-analysis showed that active tDCS was associated with greater short-term improvement in behavioral signs of consciousness in patients with DoC than sham tDCS, as reflected by an increase in the change in CRS-R score. The findings remained stable across several sensitivity analyses. Subgroup analyses suggested that treatment effects may differ by etiology and residual network modifiability, with clearer signals of benefit in patients with TBI and CVA than in those with HIBI, although this finding requires confirmation. The current evidence suggests that tDCS may be considered a potential adjunctive neuromodulatory strategy within comprehensive rehabilitation for DoC, but is insufficient to support non-selective application across all patients with DoC. The clinical value of tDCS in DoC may depend on identifying patients with preserved and modifiable consciousness-related networks and delivering more precise, individualized interventions accordingly.

## Data Availability

The original contributions presented in the study are included in the article/Supplementary material, further inquiries can be directed to the corresponding authors.
