# Supplementary material for: Efficacy of transcranial direct current stimulation for disorders of consciousness: an updated systematic review and meta-analysis based on sham-controlled randomized trials
Source: Front Neurosci. 2026 Aug 4;20:1918788. doi: 10.3389/fnins.2026.1918788 (PMC13481543; doi:10.3389/fnins.2026.1918788)
Supplement: Supplementary file 1 [file Data_Sheet_1.PDF]

Supplementary Appendix

Efficacy of transcranial direct current stimulation for disorders of consciousness: an updated systematic review and meta-analysis based on sham-controlled randomized trials

Table of contents

Appendix E1: PRISMA 2020 checklist 1

Appendix E2: Search strategies 5

Appendix E3: Data conversion methods for CRS-R change scores 14

Appendix E4: Characteristics and stimulation parameters of included studies 15

Appendix E5: Risk of bias assessment 18

Appendix E6: Subgroup analyses for CRS-R change scores 20

Appendix E7: Sensitivity analyses for the primary outcome 25

Appendix E8: Publication bias and small-study effects for the primary analysis 28

Appendix E9: GRADE summary of findings and certainty of evidence 29

Appendix E10: References and analysis inclusion status of included studies 30

Appendix E1 PRISMA 2020 checklist

Table E1.1. PRISMA 2020 checklist

Section and Topic	Item #	Checklist item	Location where item is reported
**TITLE**			
Title	1	Identify the report as a systematic review.	P1 (Title)
**ABSTRACT**			
Abstract	2	See the PRISMA 2020 for Abstracts checklist.	P2-3 (Abstract)
**INTRODUCTION**			
Rationale	3	Describe the rationale for the review in the context of existing knowledge.	P2–3 (Introduction)
Objectives	4	Provide an explicit statement of the objective(s) or question(s) the review addresses.	P2-3 (Introduction)
**METHODS**			
Eligibility criteria	5	Specify the inclusion and exclusion criteria for the review and how studies were grouped for the syntheses.	P4–6 (Methods, Eligibility criteria and Statistical analysis)
Information sources	6	Specify all databases, registers, websites, organisations, reference lists and other sources searched or consulted to identify studies. Specify the date when each source was last searched or consulted.	P4 (Methods, Search strategy)
Search strategy	7	Present the full search strategies for all databases, registers and websites, including any filters and limits used.	P4 (Methods, Search strategy); Appendix E2
Selection process	8	Specify the methods used to decide whether a study met the inclusion criteria of the review, including how many reviewers screened each record and each report retrieved, whether they worked independently, and if applicable, details of automation tools used in the process.	P4–5 (Methods, Study selection and data extraction)
Data collection process	9	Specify the methods used to collect data from reports, including how many reviewers collected data from each report, whether they worked independently, any processes for obtaining or confirming data from study investigators, and if applicable, details of automation tools used in the process.	P4–5 (Methods, Study selection and data extraction)
Data items	10a	List and define all outcomes for which data were sought. Specify whether all results that were compatible with each outcome domain in each study were sought (e.g. for all measures, time points, analyses), and if not, the methods used to decide which results to collect.	P4–5 (Methods, Study selection and data extraction); Appendix E3

Section and Topic	Item #	Checklist item	Location where item is reported
	10b	List and define all other variables for which data were sought (e.g. participant and intervention characteristics, funding sources). Describe any assumptions made about any missing or unclear information.	P4–6 (Methods, Study selection and data extraction; Statistical analysis); Appendix E4
Study risk of bias assessment	11	Specify the methods used to assess risk of bias in the included studies, including details of the tool(s) used, how many reviewers assessed each study and whether they worked independently, and if applicable, details of automation tools used in the process.	P5 (Methods, Risk of bias and certainty of evidence)
Effect measures	12	Specify for each outcome the effect measure(s) (e.g. risk ratio, mean difference) used in the synthesis or presentation of results.	P5–6 (Methods, Statistical analysis)
Synthesis methods	13a	Describe the processes used to decide which studies were eligible for each synthesis (e.g. tabulating the study intervention characteristics and comparing against the planned groups for each synthesis (item #5)).	P4–6 (Methods, Eligibility criteria and Statistical analysis); Appendix E10
	13b	Describe any methods required to prepare the data for presentation or synthesis, such as handling of missing summary statistics, or data conversions.	P4-5 (Methods, Study selection and data extraction); Appendix E3
	13c	Describe any methods used to tabulate or visually display results of individual studies and syntheses.	P6–13 (Results, Tables and Figures); Appendix E6–E8
	13d	Describe any methods used to synthesize results and provide a rationale for the choice(s). If meta-analysis was performed, describe the model(s), method(s) to identify the presence and extent of statistical heterogeneity, and software package(s) used.	P5–6 (Methods, Statistical analysis)
	13e	Describe any methods used to explore possible causes of heterogeneity among study results (e.g. subgroup analysis, meta-regression).	P5–6 (Methods, Statistical analysis)
	13f	Describe any sensitivity analyses conducted to assess robustness of the synthesized results.	P5–6 (Methods, Statistical analysis)
Reporting bias assessment	14	Describe any methods used to assess risk of bias due to missing results in a synthesis (arising from reporting biases).	P5–6 (Methods, Statistical analysis)
Certainty assessment	15	Describe any methods used to assess certainty (or confidence) in the body of evidence for an outcome.	P5 (Methods, Risk of bias and certainty of evidence)
**RESULTS**			
Study selection	16a	Describe the results of the search and selection process, from the number of records identified in the search to the number of studies included in the review, ideally using a flow diagram.	P6 (Results, Study selection; Figures 1)

Section and Topic	Item #	Checklist item	Location where item is reported
	16b	Cite studies that might appear to meet the inclusion criteria, but which were excluded, and explain why they were excluded.	P6 (Results, Study selection; Figures 1)
Study characteristics	17	Cite each included study and present its characteristics.	P7–8 (Results, Characteristics of included studies; Table 1); Appendix E4
Risk of bias in studies	18	Present assessments of risk of bias for each included study.	P9–11 (Results, Risk of bias assessment; Figures 2–3); Appendix E5
Results of individual studies	19	For all outcomes, present, for each study: (a) summary statistics for each group (where appropriate) and (b) an effect estimate and its precision (e.g. confidence/credible interval), ideally using structured tables or plots.	P11 (Results, Figures 4-5); Appendix E4 and E6
Results of syntheses	20a	For each synthesis, briefly summarise the characteristics and risk of bias among contributing studies.	P7–11 (Results, Characteristics of included studies; Risk of bias assessment; Effect of tDCS on change in CRS-R score)
	20b	Present results of all statistical syntheses conducted. If meta-analysis was done, present for each the summary estimate and its precision (e.g. confidence/credible interval) and measures of statistical heterogeneity. If comparing groups, describe the direction of the effect.	P11–14 (Results, Effect of tDCS on change in CRS-R score; Subgroup analyses; Figures 4-5 and Table 2); Appendix E6
	20c	Present results of all investigations of possible causes of heterogeneity among study results.	P12–14 (Results, Subgroup analyses; igures 5 andTable 2); Appendix E6
	20d	Present results of all sensitivity analyses conducted to assess the robustness of the synthesized results.	P15 (Results, Sensitivity analyses); Appendix E7
Reporting biases	21	Present assessments of risk of bias due to missing results (arising from reporting biases) for each synthesis assessed.	P15 (Results, Publication bias and small-study effects; Figures 6); Appendix E8
Certainty of evidence	22	Present assessments of certainty (or confidence) in the body of evidence for each outcome assessed.	P16 (Results, GRADE certainty of evidence); Appendix E9
**DISCUSSION**			
Discussion	23a	Provide a general interpretation of the results in the context of other evidence.	P16–20 (Discussion)
	23b	Discuss any limitations of the evidence included in the review.	P20 (Strengths and limitations)

Section and Topic	Item #	Checklist item	Location where item is reported
	23c	Discuss any limitations of the review processes used.	P20 (Strengths and limitations)
	23d	Discuss implications of the results for practice, policy, and future research.	P19–20 (Clinical implications and safety; Strengths and limitations)
**OTHER INFORMATION**			
Registration and protocol	24a	Provide registration information for the review, including register name and registration number, or state that the review was not registered.	P1-2 (Abstract); P4 (Methods)
	24b	Indicate where the review protocol can be accessed, or state that a protocol was not prepared.	P4 (Methods)
	24c	Describe and explain any amendments to information provided at registration or in the protocol.	No amendments to the registered protocol were made.
Support	25	Describe sources of financial or non-financial support for the review, and the role of the funders or sponsors in the review.	P21 (Funding)
Competing interests	26	Declare any competing interests of review authors.	P21 (Competing interests)
Availability of data, code and other materials	27	Report which of the following are publicly available and where they can be found: template data collection forms; data extracted from included studies; data used for all analyses; analytic code; any other materials used in the review.	P21 (Data availability)

Appendix E2: Search strategies

Electronic databases: PubMed, Embase, Cochrane Library, Scopus, and Web of Science.

Table E2.1. Search strategy for PubMed

#	**Searches**
1	"Consciousness Disorders"[Mesh]
2	((((((((Consciousness Disorder[Title/Abstract]) OR (Disorder of Consciousness[Title/Abstract])) OR (Disorders of Consciousness[Title/Abstract])) OR (Consciousness, Level Depressed[Title/Abstract])) OR (Depressed Level of Consciousness[Title/Abstract])) OR (Consciousness, Level Altered[Title/Abstract])) OR (Altered Level of Consciousness[Title/Abstract])) OR (Semiconsciousness[Title/Abstract])) OR (DOC[Title/Abstract])
3	#1 OR #2
4	"Persistent Vegetative State"[Mesh]
5	((Persistent Unawareness State[Title/Abstract]) OR (Persistent Unawareness States[Title/Abstract])) OR (State, Persistent Unawareness[Title/Abstract])) OR (States, Persistent Unawareness[Title/Abstract])) OR (Unawareness State, Persistent[Title/Abstract])) OR (Unawareness States, Persistent[Title/Abstract])) OR (PVS (Persistent Vegetative State[Title/Abstract]))) OR (PVSs (Persistent Vegetative State[Title/Abstract]))) OR (Vegetative State, Persistent[Title/Abstract])) OR (Persistent Vegetative States[Title/Abstract])) OR (State, Persistent Vegetative[Title/Abstract])) OR (States, Persistent Vegetative[Title/Abstract])) OR (Vegetative States, Persistent[Title/Abstract])) OR (Vegetative State[Title/Abstract])) OR (States, Vegetative[Title/Abstract])) OR (State, Vegetative[Title/Abstract])) OR (Vegetative States[Title/Abstract])) OR (Minimally Conscious State[Title/Abstract])) OR (Minimally Conscious States[Title/Abstract])) OR (State, Minimally Conscious[Title/Abstract])) OR (States, Minimally Conscious[Title/Abstract])) OR (Permanent Vegetative State[Title/Abstract])) OR (Permanent Vegetative States[Title/Abstract])) OR (State, Permanent Vegetative[Title/Abstract])) OR (States, Permanent Vegetative[Title/Abstract])) OR (Vegetative State, Permanent[Title/Abstract])) OR (Vegetative States, Permanent[Title/Abstract])) OR (Post-Traumatic Vegetative State[Title/Abstract])) OR (Post Traumatic Vegetative State[Title/Abstract])) OR (Post-Traumatic Vegetative States[Title/Abstract])) OR (State, Post-Traumatic Vegetative[Title/Abstract])) OR (States, Post-Traumatic Vegetative[Title/Abstract])) OR (Vegetative State, Post-Traumatic[Title/Abstract])) OR (Vegetative States, Post-Traumatic[Title/Abstract])) OR (Post-Traumatic Unawareness State[Title/Abstract])) OR (Post Traumatic Unawareness State[Title/Abstract])) OR (Post-Traumatic Unawareness States[Title/Abstract])) OR (State, Post-Traumatic Unawareness[Title/Abstract])) OR (States, Post-Traumatic Unawareness[Title/Abstract])) OR (Unawareness State, Post-Traumatic[Title/Abstract])) OR (Unawareness States, Post-Traumatic[Title/Abstract])) OR (Posttraumatic Unawareness State[Title/Abstract])) OR (Posttraumatic Unawareness States[Title/Abstract])) OR (State, Posttraumatic Unawareness[Title/Abstract])) OR (States, Posttraumatic

	Unawareness[Title/Abstract])) OR (Unawareness State, Posttraumatic[Title/Abstract])) OR (Unawareness States, Posttraumatic[Title/Abstract])) OR (Post-Comatose Unawareness State[Title/Abstract])) OR (Post Comatose Unawareness State[Title/Abstract])) OR (Post-Comatose Unawareness States[Title/Abstract])) OR (State, Post-Comatose Unawareness[Title/Abstract])) OR (States, Post-Comatose Unawareness[Title/Abstract])) OR (Unawareness State, Post-Comatose[Title/Abstract])) OR (Unawareness States, Post-Comatose[Title/Abstract])) OR (Postcomatose Unawareness State[Title/Abstract])) OR (Postcomatose Unawareness States[Title/Abstract])) OR (State, Postcomatose Unawareness[Title/Abstract])) OR (States, Postcomatose Unawareness[Title/Abstract])) OR (Unawareness State, Postcomatose[Title/Abstract])) OR (Unawareness States, Postcomatose[Title/Abstract])) OR (Prolonged Post-Traumatic Unawareness[Title/Abstract])) OR (Post-Traumatic Unawarenesses, Prolonged[Title/Abstract])) OR (Post-Traumatic Unawareness, Prolonged[Title/Abstract])) OR (Prolonged Post Traumatic Unawareness[Title/Abstract])) OR (Prolonged Post-Traumatic Unawarenesses[Title/Abstract])) OR (Unawarenesses, Prolonged Post-Traumatic[Title/Abstract])) OR (Unawareness, Prolonged Post-Traumatic[Title/Abstract])) OR (Transient Vegetative State[Title/Abstract])) OR (States, Transient Vegetative[Title/Abstract])) OR (State, Transient Vegetative[Title/Abstract])) OR (Transient Vegetative States[Title/Abstract])) OR (Vegetative States, Transient[Title/Abstract])) OR (Vegetative State, Transient[Title/Abstract])) OR (unresponsive wakefulness syndrome[Title/Abstract])) OR (UWS[Title/Abstract])
6	#4 OR #5
7	"Coma, Post-Head Injury"[Mesh]
8	((((((((((((((((((Coma, Post Head Injury[Title/Abstract]) OR (Comas, Post-Head Injury[Title/Abstract])) OR (Post-Head Injury Comas[Title/Abstract])) OR (Coma, Traumatic[Title/Abstract])) OR (Traumatic Coma[Title/Abstract])) OR (Post-Head Injury Coma[Title/Abstract])) OR (Post Head Injury Coma[Title/Abstract])) OR (Coma, Post-Trauma[Title/Abstract])) OR (Coma, Post Trauma[Title/Abstract])) OR (Comas, Post-Trauma[Title/Abstract])) OR (Post-Trauma Coma[Title/Abstract])) OR (Post-Trauma Comas[Title/Abstract])) OR (Coma, Post-Traumatic[Title/Abstract])) OR (Coma, Post Traumatic[Title/Abstract])) OR (Post-Traumatic Coma[Title/Abstract])) OR (Coma, Post-Traumatic, Prolonged[Title/Abstract])) OR (Coma, Post-Concussive[Title/Abstract])) OR (Coma, Post Concussive[Title/Abstract])) OR (Comas, Post-Concussive[Title/Abstract])) OR (Post-Concussive Coma[Title/Abstract])) OR (Post-Concussive Comas[Title/Abstract])
9	#7 OR #8
10	#3 OR #6 OR #9
11	"Transcranial Direct Current Stimulation"[Mesh]
12	(((((((((((((((((((tDCS[Title/Abstract]) OR (Anodal Stimulation Transcranial Direct Current Stimulation[Title/Abstract])) OR (Anodal Stimulation tDCS[Title/Abstract])) OR (Anodal Stimulation tDCSs[Title/Abstract])) OR (Stimulation tDCS, Anodal[Title/Abstract])) OR (Stimulation tDCSs, Anodal[Title/Abstract])) OR (tDCS, Anodal Stimulation[Title/Abstract])) OR (tDCSs, Anodal Stimulation[Title/Abstract])) OR (Cathodal Stimulation Transcranial Direct Current Stimulation[Title/Abstract])) OR (Cathodal Stimulation tDCS[Title/Abstract])) OR (Cathodal Stimulation tDCSs[Title/Abstract])) OR (Stimulation tDCS, Cathodal[Title/Abstract])) OR (Stimulation tDCSs, Cathodal[Title/Abstract])) OR (tDCS,

	Cathodal Stimulation[Title/Abstract])) OR (tDCSs, Cathodal Stimulation[Title/Abstract])) OR (Repetitive Transcranial Electrical Stimulation[Title/Abstract])) OR (Transcranial Electrical Stimulation[Title/Abstract])) OR (Electrical Stimulations, Transcranial[Title/Abstract])) OR (Electrical Stimulation, Transcranial[Title/Abstract])) OR (Stimulations, Transcranial Electrical[Title/Abstract])) OR (Stimulation, Transcranial Electrical[Title/Abstract])) OR (Transcranial Electrical Stimulations[Title/Abstract])
13	#11 OR #12
14	((randomized controlled trial[Publication Type] OR controlled clinical trial[Publication Type] OR randomized[Title/Abstract] OR randomised[Title/Abstract] OR randomly[Title/Abstract] OR placebo[Title/Abstract]) OR (RCT[Title/Abstract])) OR (RCTs[Title/Abstract])
15	#10 AND #13 AND #14

Table E2.2. Search strategy for Embase

#	**Searches**
1	'coma'/exp OR 'coma'
2	'Coma, Post Head Injury':ab,ti OR 'Comas, Post-Head Injury':ab,ti OR 'Post-Head Injury Comas':ab,ti OR 'Coma, Traumatic':ab,ti OR 'Traumatic Coma':ab,ti OR 'Post-Head Injury Coma':ab,ti OR 'Post Head Injury Coma':ab,ti OR 'Coma, Post-Trauma':ab,ti OR 'Coma, Post Trauma':ab,ti OR 'Comas, Post-Trauma':ab,ti OR 'Post-Trauma Coma':ab,ti OR 'Post-Trauma Comas':ab,ti OR 'Coma, Post-Traumatic':ab,ti OR 'Coma, Post Traumatic':ab,ti OR 'Post-Traumatic Coma':ab,ti OR 'Coma, Post-Traumatic, Prolonged':ab,ti OR 'Coma, Post-Concussive':ab,ti OR 'Coma, Post Concussive':ab,ti OR 'Comas, Post-Concussive':ab,ti OR 'Post-Concussive Coma':ab,ti OR 'Post-Concussive Comas':ab,ti
3	#1 OR #2
4	'consciousness disorder'/exp OR 'consciousness disorder'
5	'Consciousness Disorder':ab,ti OR 'Disorder of Consciousness':ab,ti OR 'Disorders of Consciousness':ab,ti OR 'Consciousness, Level Depressed':ab,ti OR 'Depressed Level of Consciousness':ab,ti OR 'Consciousness, Level Altered':ab,ti OR 'Altered Level of Consciousness':ab,ti OR 'Semiconsciousness':ab,ti
6	#4 OR #5
7	'persistent vegetative state'/exp OR 'persistent vegetative state'
8	'Persistent Unawareness State':ab,ti OR 'Persistent Unawareness States':ab,ti OR 'State, Persistent Unawareness':ab,ti OR 'States, Persistent Unawareness':ab,ti OR 'Unawareness State, Persistent':ab,ti OR 'Unawareness States, Persistent':ab,ti OR 'PVS (Persistent Vegetative State)':ab,ti OR 'PVSs (Persistent Vegetative State)':ab,ti OR 'Vegetative State, Persistent':ab,ti OR 'Persistent Vegetative States':ab,ti OR 'State, Persistent Vegetative':ab,ti OR 'States, Persistent Vegetative':ab,ti OR 'Vegetative States, Persistent':ab,ti OR 'Vegetative State':ab,ti OR 'States, Vegetative':ab,ti OR 'State, Vegetative':ab,ti OR 'Vegetative States':ab,ti OR

	'Minimally Conscious State':ab,ti OR 'Minimally Conscious States':ab,ti OR 'State, Minimally Conscious':ab,ti OR 'States, Minimally Conscious':ab,ti OR 'Permanent Vegetative State':ab,ti OR 'Permanent Vegetative States':ab,ti OR 'State, Permanent Vegetative':ab,ti OR 'States, Permanent Vegetative':ab,ti OR 'Vegetative State, Permanent':ab,ti OR 'Vegetative States, Permanent':ab,ti OR 'Post-Traumatic Vegetative State':ab,ti OR 'Post Traumatic Vegetative State':ab,ti OR 'Post-Traumatic Vegetative States':ab,ti OR 'State, Post-Traumatic Vegetative':ab,ti OR 'States, Post-Traumatic Vegetative':ab,ti OR 'Vegetative State, Post-Traumatic':ab,ti OR 'Vegetative States, Post-Traumatic':ab,ti OR 'Post-Traumatic Unawareness State':ab,ti OR 'Post Traumatic Unawareness State':ab,ti OR 'Post-Traumatic Unawareness States':ab,ti OR 'State, Post-Traumatic Unawareness':ab,ti OR 'States, Post-Traumatic Unawareness':ab,ti OR 'Unawareness State, Post-Traumatic':ab,ti OR 'Unawareness States, Post-Traumatic':ab,ti OR 'Posttraumatic Unawareness State':ab,ti OR 'Posttraumatic Unawareness States':ab,ti OR 'State, Posttraumatic Unawareness':ab,ti OR 'States, Posttraumatic Unawareness':ab,ti OR 'Unawareness State, Posttraumatic':ab,ti OR 'Unawareness States, Posttraumatic':ab,ti OR 'Post-Comatose Unawareness State':ab,ti OR 'Post Comatose Unawareness State':ab,ti OR 'Post-Comatose Unawareness States':ab,ti OR 'State, Post-Comatose Unawareness':ab,ti OR 'States, Post-Comatose Unawareness':ab,ti OR 'Unawareness State, Post-Comatose':ab,ti OR 'Unawareness States, Post-Comatose':ab,ti OR 'Postcomatose Unawareness State':ab,ti OR 'Postcomatose Unawareness States':ab,ti OR 'State, Postcomatose Unawareness':ab,ti OR 'States, Postcomatose Unawareness':ab,ti OR 'Unawareness State, Postcomatose':ab,ti OR 'Unawareness States, Postcomatose':ab,ti OR 'Prolonged Post-Traumatic Unawareness':ab,ti OR 'Post-Traumatic Unawarenesses, Prolonged':ab,ti OR 'Post-Traumatic Unawareness, Prolonged':ab,ti OR 'Prolonged Post Traumatic Unawareness':ab,ti OR 'Prolonged Post-Traumatic Unawarenesses':ab,ti OR 'Unawarenesses, Prolonged Post-Traumatic':ab,ti OR 'Unawareness, Prolonged Post-Traumatic':ab,ti OR 'Transient Vegetative State':ab,ti OR 'States, Transient Vegetative':ab,ti OR 'State, Transient Vegetative':ab,ti OR 'Transient Vegetative States':ab,ti OR 'Vegetative States, Transient':ab,ti OR 'Vegetative State, Transient':ab,ti OR 'unresponsive wakefulness syndrome':ab,ti OR 'UWS':ab,ti
9	#7 OR #8
10	#3 OR #6 OR #9
11	'transcranial direct current stimulation'/exp OR 'transcranial direct current stimulation'
12	'tDCS':ab,ti OR 'Anodal Stimulation Transcranial Direct Current Stimulation':ab,ti OR 'Anodal Stimulation tDCS':ab,ti OR 'Anodal Stimulation tDCSs':ab,ti OR 'Stimulation tDCS, Anodal':ab,ti OR 'Stimulation tDCSs, Anodal':ab,ti OR 'tDCS, Anodal Stimulation':ab,ti OR 'tDCSs, Anodal Stimulation':ab,ti OR 'Cathodal Stimulation Transcranial Direct Current Stimulation':ab,ti OR 'Cathodal Stimulation tDCS':ab,ti OR 'Cathodal Stimulation tDCSs':ab,ti OR 'Stimulation tDCS, Cathodal':ab,ti OR 'Stimulation tDCSs, Cathodal':ab,ti OR 'tDCS, Cathodal Stimulation':ab,ti OR 'tDCSs, Cathodal Stimulation':ab,ti OR 'Repetitive Transcranial Electrical Stimulation':ab,ti OR 'Transcranial Electrical Stimulation':ab,ti OR 'Electrical Stimulations, Transcranial':ab,ti OR 'Electrical Stimulation, Transcranial':ab,ti OR

	'Stimulations, Transcranial Electrical':ab,ti OR 'Stimulation, Transcranial Electrical':ab,ti OR 'Transcranial Electrical Stimulations':ab,ti
13	#11 OR #12
14	'randomized controlled trial':ab,ti OR 'randomized':ab,ti OR 'placebo':ab,ti OR 'RCT':ab,ti
15	#10 AND #13 AND #14

Table E2.3. Search strategy for Web of Science

#	**Searches**
1	TS=(Coma, Post-Head Injury OR Coma, Post Head Injury OR Comas, Post-Head Injury OR Post-Head Injury Comas OR Coma, Traumatic OR Traumatic Coma OR Post-Head Injury Coma OR Post Head Injury Coma OR Coma, Post-Trauma OR Coma, Post Trauma OR Comas, Post-Trauma OR Post-Trauma Coma OR Post-Trauma Comas OR Coma, Post-Traumatic OR Coma, Post Traumatic OR Post-Traumatic Coma OR Coma, Post-Traumatic, Prolonged OR Coma, Post-Concussive OR Coma, Post Concussive OR Comas, Post-Concussive OR Post-Concussive Coma OR Post-Concussive Comas)
2	TS=(Consciousness Disorders OR Consciousness Disorder OR Disorder of Consciousness OR Disorders of Consciousness OR Consciousness, Level Depressed OR Depressed Level of Consciousness OR Consciousness, Level Altered OR Altered Level of Consciousness OR Semiconsciousness)
3	TS=(Persistent Vegetative State OR Persistent Unawareness State OR Persistent Unawareness States OR State, Persistent Unawareness OR States, Persistent Unawareness OR Unawareness State, Persistent OR Unawareness States, Persistent OR PVS (Persistent Vegetative State) OR PVSs (Persistent Vegetative State) OR Vegetative State, Persistent OR Persistent Vegetative States OR State, Persistent Vegetative OR States, Persistent Vegetative OR Vegetative States, Persistent OR Vegetative State OR States, Vegetative OR State, Vegetative OR Vegetative States OR Minimally Conscious State OR Minimally Conscious States OR State, Minimally Conscious OR States, Minimally Conscious OR Permanent Vegetative State OR Permanent Vegetative States OR State, Permanent Vegetative OR States, Permanent Vegetative OR Vegetative State, Permanent OR Vegetative States, Permanent OR Post-Traumatic Vegetative State OR Post Traumatic Vegetative State OR Post-Traumatic Vegetative States OR State, Post-Traumatic Vegetative OR States, Post-Traumatic Vegetative OR Vegetative State, Post-Traumatic OR Vegetative States, Post-Traumatic OR Post-Traumatic Unawareness State OR Post Traumatic Unawareness State OR Post-Traumatic Unawareness States OR State, Post-Traumatic Unawareness OR States, Post-Traumatic Unawareness OR Unawareness State, Post-Traumatic OR Unawareness States, Post-Traumatic OR Posttraumatic Unawareness State OR Posttraumatic Unawareness States OR State, Posttraumatic Unawareness OR States, Posttraumatic Unawareness OR Unawareness State, Posttraumatic OR Unawareness States, Posttraumatic OR Post-Comatose Unawareness State OR Post Comatose Unawareness State OR Post-Comatose Unawareness States OR State, Post-Comatose Unawareness OR States,

	Post-Comatose Unawareness OR Unawareness State, Post-Comatose OR Unawareness States, Post-Comatose OR Postcomatose Unawareness State OR Postcomatose Unawareness States OR State, Postcomatose Unawareness OR States, Postcomatose Unawareness OR Unawareness State, Postcomatose OR Unawareness States, Postcomatose OR Prolonged Post-Traumatic Unawareness OR Post-Traumatic Unawarenesses, Prolonged OR Post-Traumatic Unawareness, Prolonged OR Prolonged Post Traumatic Unawareness OR Prolonged Post-Traumatic Unawarenesses OR Unawarenesses, Prolonged Post-Traumatic OR Unawareness, Prolonged Post-Traumatic OR Transient Vegetative State OR States, Transient Vegetative OR State, Transient Vegetative OR Transient Vegetative States OR Vegetative States, Transient OR Vegetative State, Transient OR unresponsive wakefulness syndrome OR UWS)
4	#1 OR #2 OR #3
5	TS=(transcranial direct current stimulation OR tDCS OR Anodal Stimulation Transcranial Direct Current Stimulation OR Anodal Stimulation tDCS OR Anodal Stimulation tDCSs OR Stimulation tDCS, Anodal OR Stimulation tDCSs, Anodal OR tDCS, Anodal Stimulation OR tDCSs, Anodal Stimulation OR Cathodal Stimulation Transcranial Direct Current Stimulation OR Cathodal Stimulation tDCS OR Cathodal Stimulation tDCSs OR Stimulation tDCS, Cathodal OR Stimulation tDCSs, Cathodal OR tDCS, Cathodal Stimulation OR tDCSs, Cathodal Stimulation OR Repetitive Transcranial Electrical Stimulation OR Transcranial Electrical Stimulation OR Electrical Stimulations, Transcranial OR Electrical Stimulation, Transcranial OR Stimulations, Transcranial Electrical OR Stimulation, Transcranial Electrical OR Transcranial Electrical Stimulations)
6	AB=(randomized controlled trial OR randomized OR placebo OR RCT)
7	#4 AND #5 AND #6

Table E2.4. Search strategy for Cochrane Library

#	Searches
1	MeSH descriptor: [Coma] explode all trees
2	(Coma, Post Head Injury):ti,ab,kw OR (Comas, Post-Head Injury):ti,ab,kw OR (Post-Head Injury Comas):ti,ab,kw OR (Coma, Traumatic):ti,ab,kw OR (Traumatic Coma):ti,ab,kw OR (Post-Head Injury Coma):ti,ab,kw OR (Post Head Injury Coma):ti,ab,kw OR (Coma, Post-Trauma):ti,ab,kw OR (Coma, Post Trauma):ti,ab,kw OR (Comas, Post-Trauma):ti,ab,kw OR (Post-Trauma Coma):ti,ab,kw OR (Post-Trauma Comas):ti,ab,kw OR (Coma, Post-Traumatic):ti,ab,kw OR (Coma, Post Traumatic):ti,ab,kw OR (Post-Traumatic Coma):ti,ab,kw OR (Coma, Post-Traumatic, Prolonged):ti,ab,kw OR (Coma, Post-Concussive):ti,ab,kw OR (Coma, Post Concussive):ti,ab,kw OR (Comas, Post-Concussive):ti,ab,kw OR (Post-Concussive Coma):ti,ab,kw OR (Post-Concussive Comas):ti,ab,kw
3	#1 OR #2
4	MeSH descriptor: [Consciousness Disorders] explode all trees

5	(Consciousness Disorder):ti,ab,kw OR (Disorder of Consciousness):ti,ab,kw OR (Disorders of Consciousness):ti,ab,kw OR (Consciousness, Level Depressed):ti,ab,kw OR (Depressed Level of Consciousness):ti,ab,kw OR (Consciousness, Level Altered):ti,ab,kw OR (Altered Level of Consciousness):ti,ab,kw OR (Semiconsciousness):ti,ab,kw
6	#4 OR #5
7	MeSH descriptor: [Persistent Vegetative State] explode all trees
8	(Persistent Unawareness State):ti,ab,kw OR (Persistent Unawareness States):ti,ab,kw OR (State, Persistent Unawareness):ti,ab,kw OR (States, Persistent Unawareness):ti,ab,kw OR (Unawareness State, Persistent):ti,ab,kw OR (Unawareness States, Persistent):ti,ab,kw OR (PVS (Persistent Vegetative State)):ti,ab,kw OR (PVSs (Persistent Vegetative State)):ti,ab,kw OR (Vegetative State, Persistent):ti,ab,kw OR (Persistent Vegetative States):ti,ab,kw OR (State, Persistent Vegetative):ti,ab,kw OR (States, Persistent Vegetative):ti,ab,kw OR (Vegetative States, Persistent):ti,ab,kw OR (Vegetative State):ti,ab,kw OR (States, Vegetative):ti,ab,kw OR (State, Vegetative):ti,ab,kw OR (Vegetative States):ti,ab,kw OR (Minimally Conscious State):ti,ab,kw OR (Minimally Conscious States):ti,ab,kw OR (State, Minimally Conscious):ti,ab,kw OR (States, Minimally Conscious):ti,ab,kw OR (Permanent Vegetative State):ti,ab,kw OR (Permanent Vegetative States):ti,ab,kw OR (State, Permanent Vegetative):ti,ab,kw OR (States, Permanent Vegetative):ti,ab,kw OR (Vegetative State, Permanent):ti,ab,kw OR (Vegetative States, Permanent):ti,ab,kw OR (Post-Traumatic Vegetative State):ti,ab,kw OR (Post Traumatic Vegetative State):ti,ab,kw OR (Post-Traumatic Vegetative States):ti,ab,kw OR (State, Post-Traumatic Vegetative):ti,ab,kw OR (States, Post-Traumatic Vegetative):ti,ab,kw OR (Vegetative State, Post-Traumatic):ti,ab,kw OR (Vegetative States, Post-Traumatic):ti,ab,kw OR (Post-Traumatic Unawareness State):ti,ab,kw OR (Post Traumatic Unawareness State):ti,ab,kw OR (Post-Traumatic Unawareness States):ti,ab,kw OR (State, Post-Traumatic Unawareness):ti,ab,kw OR (States, Post-Traumatic Unawareness):ti,ab,kw OR (Unawareness State, Post-Traumatic):ti,ab,kw OR (Unawareness States, Post-Traumatic):ti,ab,kw OR (Posttraumatic Unawareness State):ti,ab,kw OR (Posttraumatic Unawareness States):ti,ab,kw OR (State, Posttraumatic Unawareness):ti,ab,kw OR (States, Posttraumatic Unawareness):ti,ab,kw OR (Unawareness State, Posttraumatic):ti,ab,kw OR (Unawareness States, Posttraumatic):ti,ab,kw OR (Post-Comatose Unawareness State):ti,ab,kw OR (Post Comatose Unawareness State):ti,ab,kw OR (Post-Comatose Unawareness States):ti,ab,kw OR (State, Post-Comatose Unawareness):ti,ab,kw OR (States, Post-Comatose Unawareness):ti,ab,kw OR (Unawareness State, Post-Comatose):ti,ab,kw OR (Unawareness States, Post-Comatose):ti,ab,kw OR (Postcomatose Unawareness State):ti,ab,kw OR (Postcomatose Unawareness States):ti,ab,kw OR (State, Postcomatose Unawareness):ti,ab,kw OR (States, Postcomatose Unawareness):ti,ab,kw OR (Unawareness State, Postcomatose):ti,ab,kw OR (Unawareness States, Postcomatose):ti,ab,kw OR (Prolonged Post-Traumatic Unawareness):ti,ab,kw OR (Post-Traumatic Unawarenesses, Prolonged):ti,ab,kw OR (Post-Traumatic Unawareness, Prolonged):ti,ab,kw OR (Prolonged Post Traumatic Unawareness):ti,ab,kw OR (Prolonged Post-Traumatic Unawarenesses):ti,ab,kw OR (Unawarenesses, Prolonged Post-Traumatic):ti,ab,kw OR (Unawareness, Prolonged Post-

	Traumatic):ti,ab,kw OR (Transient Vegetative State):ti,ab,kw OR (States, Transient Vegetative):ti,ab,kw OR (State, Transient Vegetative):ti,ab,kw OR (Transient Vegetative States):ti,ab,kw OR (Vegetative States, Transient):ti,ab,kw OR (Vegetative State, Transient):ti,ab,kw OR (unresponsive wakefulness syndrome):ti,ab,kw OR (UWS):ti,ab,kw
9	#7 OR #8
10	#3 OR #6 OR #9
11	MeSH descriptor: [Transcranial Direct Current Stimulation] explode all trees
12	(tDCS):ti,ab,kw OR (Anodal Stimulation Transcranial Direct Current Stimulation):ti,ab,kw OR (Anodal Stimulation tDCS):ti,ab,kw OR (Anodal Stimulation tDCSs):ti,ab,kw OR (Stimulation tDCS, Anodal):ti,ab,kw OR (Stimulation tDCSs, Anodal):ti,ab,kw OR (tDCS, Anodal Stimulation):ti,ab,kw OR (tDCSs, Anodal Stimulation):ti,ab,kw OR (Cathodal Stimulation Transcranial Direct Current Stimulation):ti,ab,kw OR (Cathodal Stimulation tDCS):ti,ab,kw OR (Cathodal Stimulation tDCSs):ti,ab,kw OR (Stimulation tDCS, Cathodal):ti,ab,kw OR (Stimulation tDCSs, Cathodal):ti,ab,kw OR (tDCS, Cathodal Stimulation):ti,ab,kw OR (tDCSs, Cathodal Stimulation):ti,ab,kw OR (Repetitive Transcranial Electrical Stimulation):ti,ab,kw OR (Transcranial Electrical Stimulation):ti,ab,kw OR (Electrical Stimulations, Transcranial):ti,ab,kw OR (Electrical Stimulation, Transcranial):ti,ab,kw OR (Stimulations, Transcranial Electrical):ti,ab,kw OR (Stimulation, Transcranial Electrical):ti,ab,kw OR (Transcranial Electrical Stimulations):ti,ab,kw
13	#11 OR #12
14	MeSH descriptor: [Randomized Controlled Trial] explode all trees
15	(randomized controlled trial):ti,ab,kw OR (randomized):ti,ab,kw OR (placebo):ti,ab,kw OR (RCT):ti,ab,kw
16	#14 OR #15
17	#10 AND #13 AND #16

Table E2.5. Search strategy for Scopus

#	Searches
1	TITLE-ABS-KEY ("Coma, Post-Head Injury" OR "Coma, Post Head Injury" OR "Comas, Post-Head Injury" OR "Post-Head Injury Comas" OR "Coma, Traumatic" OR "Traumatic Coma" OR "Post-Head Injury Coma" OR "Post Head Injury Coma" OR "Coma, Post-Trauma" OR "Coma, Post Trauma" OR "Comas, Post-Trauma" OR "Post-Trauma Coma" OR "Post-Trauma Comas" OR "Coma, Post-Traumatic" OR "Coma, Post Traumatic" OR "Post-Traumatic Coma" OR "Coma, Post-Traumatic, Prolonged" OR "Coma, Post-Concussive" OR "Coma, Post Concussive" OR "Comas, Post-Concussive" OR "Post-Concussive Coma" OR "Post-Concussive Comas")
2	TITLE-ABS-KEY ("Consciousness Disorders" OR "Consciousness Disorder" OR "Disorder of Consciousness" OR "Disorders of Consciousness" OR "Consciousness, Level Depressed" OR "Depressed Level of Consciousness" OR "Consciousness, Level Altered" OR "Altered Level of Consciousness" OR "Semiconsciousness")

3	TITLE-ABS-KEY ("Persistent Vegetative State" OR "Persistent Unawareness State" OR "Persistent Unawareness States" OR "State, Persistent Unawareness" OR "States, Persistent Unawareness" OR "Unawareness State, Persistent" OR "Unawareness States, Persistent" OR "PVS (Persistent Vegetative State)" OR "PVSs (Persistent Vegetative State)" OR "Vegetative State, Persistent" OR "Persistent Vegetative States" OR "State, Persistent Vegetative" OR "States, Persistent Vegetative" OR "Vegetative States, Persistent" OR "Vegetative State" OR "States, Vegetative" OR "State, Vegetative" OR "Vegetative States" OR "Minimally Conscious State" OR "Minimally Conscious States" OR "State, Minimally Conscious" OR "States, Minimally Conscious" OR "Permanent Vegetative State" OR "Permanent Vegetative States" OR "State, Permanent Vegetative" OR "States, Permanent Vegetative" OR "Vegetative State, Permanent" OR "Vegetative States, Permanent" OR "Post-Traumatic Vegetative State" OR "Post Traumatic Vegetative State" OR "Post-Traumatic Vegetative States" OR "State, Post-Traumatic Vegetative" OR "States, Post-Traumatic Vegetative" OR "Vegetative State, Post-Traumatic" OR "Vegetative States, Post-Traumatic" OR "Post-Traumatic Unawareness State" OR "Post Traumatic Unawareness State" OR "Post-Traumatic Unawareness States" OR "State, Post-Traumatic Unawareness" OR "States, Post-Traumatic Unawareness" OR "Unawareness State, Post-Traumatic" OR "Unawareness States, Post-Traumatic" OR "Posttraumatic Unawareness State" OR "Posttraumatic Unawareness States" OR "State, Posttraumatic Unawareness" OR "States, Posttraumatic Unawareness" OR "Unawareness State, Posttraumatic" OR "Unawareness States, Posttraumatic" OR "Post-Comatose Unawareness State" OR "Post Comatose Unawareness State" OR "Post-Comatose Unawareness States" OR "State, Post-Comatose Unawareness" OR "States, Post-Comatose Unawareness" OR "Unawareness State, Post-Comatose" OR "Unawareness States, Post-Comatose" OR "Postcomatose Unawareness State" OR "Postcomatose Unawareness States" OR "State, Postcomatose Unawareness" OR "States, Postcomatose Unawareness" OR "Unawareness State, Postcomatose" OR "Unawareness States, Postcomatose" OR "Prolonged Post-Traumatic Unawareness" OR "Post-Traumatic Unawarenesses, Prolonged" OR "Post-Traumatic Unawareness, Prolonged" OR "Prolonged Post Traumatic Unawareness" OR "Prolonged Post-Traumatic Unawarenesses" OR "Unawarenesses, Prolonged Post-Traumatic" OR "Unawareness, Prolonged Post-Traumatic" OR "Transient Vegetative State" OR "States, Transient Vegetative" OR "State, Transient Vegetative" OR "Transient Vegetative States" OR "Vegetative States, Transient" OR "Vegetative State, Transient" OR "unresponsive wakefulness syndrome" OR "UWS")
4	#1 OR #2 OR #3
5	TITLE-ABS-KEY ("Transcranial Direct Current Stimulation" OR "tDCS" OR "Anodal Stimulation Transcranial Direct Current Stimulation" OR "Anodal Stimulation tDCS" OR "Anodal Stimulation tDCSs" OR "Stimulation tDCS, Anodal" OR "Stimulation tDCSs, Anodal" OR "tDCS, Anodal Stimulation" OR "tDCSs, Anodal Stimulation" OR "Cathodal Stimulation Transcranial Direct Current Stimulation" OR "Cathodal Stimulation tDCS" OR "Cathodal Stimulation tDCSs" OR "Stimulation tDCS, Cathodal" OR "Stimulation tDCSs, Cathodal" OR "tDCS, Cathodal Stimulation" OR "tDCSs, Cathodal Stimulation" OR "Repetitive Transcranial Electrical Stimulation" OR "Transcranial Electrical Stimulation" OR "Electrical Stimulations, Transcranial" OR "Electrical Stimulation, Transcranial" OR "Stimulations, Transcranial Electrical" OR "Stimulation, Transcranial Electrical" OR "Transcranial Electrical Stimulations")
6	TITLE-ABS-KEY ("randomized controlled trial" OR "randomized" OR "placebo" OR "RCT")
7	#4 AND #5 AND #6

Appendix E3. Data conversion methods for CRS-R change scores

The primary outcome of this study was the change in CRS-R score, defined as the post-intervention CRS-R score minus the baseline CRS-R score. The direction of effect was harmonized so that positive values indicated greater improvement in consciousness in the active tDCS group than in the sham tDCS group. When change-score means and standard deviations were directly reported in the original articles, these data were extracted preferentially. If individual-level data were provided in the article or supplementary materials, CRS-R change scores were calculated for each participant, and the corresponding group-level means and standard deviations were then derived. When change-score data were not directly reported, the following conversion rules were applied.

1. Mean change

When the mean CRS-R change score was not reported but baseline and post-intervention means were available, the mean change was calculated as:

$$Mean_{change} = Mean_{post} - Mean_{pre}$$

where Mean_post and Mean_pre denote the post-intervention and baseline CRS-R means, respectively.

2. Standard deviation of change scores

When the standard deviation of the CRS-R change score was not reported but baseline and post-intervention standard deviations were available, it was estimated as:

$$SD_{change} = \sqrt{SD_{pre}^2 + SD_{post}^2 - 2 \times r \times SD_{pre} \times SD_{post}}$$

where SD_pre and SD_post denote the baseline and post-intervention standard deviations, respectively, and (r) denotes the within-participant correlation between baseline and post-intervention measurements. Because this correlation was not reported in the original study, r = 0.5 was prespecified and applied consistently during data extraction and analysis.

3. Data handling for randomized crossover trials

For randomized crossover trials, only first-period data were extracted to minimize potential carryover effects. If individual-level CRS-R data were available for the first period, change scores were calculated for each participant and then summarized as group-level means and standard deviations. If only first-period baseline and post-intervention group-level data were available, the mean and standard deviation of change scores were calculated using the formulas above. Studies without obtainable first-period data were not included in the main analysis.

Appendix E4. Characteristics and stimulation parameters of included studies

Table E4.1. Characteristics of studies included in the quantitative synthesis

Study	Design	Group	n	Male/Female	Age, years	DoC type	Disease duration, months	Etiology	Baseline CRS-R	Stimulation target	Adverse events
Zhang 2017	Parallel RCT	Active tDCS	13	7/6	58.54±15.78	MCS 8; VS 5	5.70±4.60	Hypoxia 2; TBI 5; hemorrhage 5; ischemia 1	6.85±2.85	Left DLPFC	0
		Sham tDCS	13	8/5	46.85±22.90	MCS 7; VS 6	5.00±3.80	Hypoxia 3; TBI 7; hemorrhage 2; ischemia 1	7.77±3.09	Sham stimulation	0
Yu 2022	Parallel RCT	Active tDCS	15	15/0	51.73±9.40	VS 15	6.35±5.42	Stroke 15	5.80±1.42	Left DLPFC	NR
		Sham tDCS	16	13/3	59.38±9.50	VS 16	6.00±3.21	Stroke 16	5.50±1.37	Sham stimulation	NR
Wu 2019	Parallel RCT, three-arm trial	Active tDCS-left DLPFC	5	4/1	44.40±27.25	MCS 3; UWS 2	152.40±98.71	Hemorrhage 3; trauma 2	7.80±3.27	Left DLPFC	0
		Active tDCS-right DLPFC	5	2/3	54.80±12.68	MCS 1; UWS 4	227.80±234.75	Hemorrhage 4; trauma 1	5.80±3.56	Right DLPFC	0
		Sham tDCS	5	3/2	44.40±10.95	MCS 3; UWS 2	78.20±58.42	Anoxia 3; trauma 2	7.80±3.63	Sham stimulation	0
Thibaut 2017	Crossover RCT	Active tDCS	9	5/4	40.89±11.24	MCS 9	94.00±113.37	TBI 6; cardiac arrest 2; anoxic stroke 1	8.89±3.10	Left DLPFC	Skin redness, 4 cases; not reported by group
		Sham tDCS	7	4/3	46.43±20.58	MCS 7	58.00±86.18	TBI 5; cardiac arrest 1; anoxic stroke 1	8.86±3.67	Sham stimulation	Skin redness, 4 cases; not reported by group
Thibaut 2023	Parallel RCT	Active tDCS	29	21/8	41.10±15.33	MCS 13; UWS/VS 16	10.09±6.71	TBI 12; anoxic/hypoxic 14; CVA/vascular 3	8.62±3.91	Left DLPFC	Skin redness, 3 cases; skin irritation, 1 case; somnolence, 1 case; not reported by group
		Sham tDCS	23	14/9	44.61±13.41	MCS 11; UWS/VS 12	7.11±5.19	TBI 7; anoxic/hypoxic 9; CVA/vascular 7	7.74±3.89	Sham stimulation	Skin redness, 3 cases; skin irritation, 1 case; somnolence, 1 case; not reported by group
Martens 2018	Crossover RCT	Active tDCS	16	10/6	47.56±15.71	MCS 16	79.00±63.18	TBI 3; ischemic stroke 2; cardiac arrest 7; aneurysm 3; anoxia 1	9.25±4.42	Left DLPFC	Skin redness, 7 cases; somnolence, 4 cases
		Sham tDCS	11	9/2	33.91±7.25	MCS 11	122.91±103.75	TBI 9; cardiac arrest 2	8.91±2.81	Sham stimulation	Skin redness, 5 cases; somnolence, 1 case

Martens 2020	Crossover RCT	Active tDCS	24	15/9	48.75±13.63	MCS 16; UWS/VS 8	53.71±83.98	TBI 12; non-TBI 12	8.21±3.49	Bilateral frontoparietal network	0
		Sham tDCS	16	9/7	43.81±16.31	MCS 7; UWS/VS 9	14.86±17.36	TBI 4; non-TBI 12	6.69±3.36	Sham stimulation	0
Martens 2019	Crossover RCT	Active tDCS	4	2/2	53.25±21.28	MCS 2; UWS/VS 2	3.11±4.21	TBI 2; non-TBI 2	8.25±6.65	Primary motor cortex	0
		Sham tDCS	6	6/0	46.33±25.07	MCS 4; UWS/VS 2	9.84±16.81	TBI 3; non-TBI 3	8.33±6.09	Sham stimulation	0
Huang 2017	Crossover RCT	Active tDCS	15	10/5	54.33±13.24	MCS 15	6.60±5.82	Trauma 7; CVA 6; subarachnoid hemorrhage 1; hypoxic-ischemic 1	12.00±3.32	Posterior parietal cortex	0
		Sham tDCS	18	10/8	59.11±8.41	MCS 18	4.94±3.64	Trauma 13; CVA 4; hypoxic-ischemic 1	11.06±3.06	Sham stimulation	0
Estraneo 2017	Crossover RCT	Active tDCS	8	6/2	54.13±23.79	VS 5; MCS 3	16.88±27.47	Traumatic 1; vascular 3; anoxic 4	6.88±3.76	Left DLPFC	0
		Sham tDCS	5	1/4	55.20±20.32	VS 2; MCS 3	25.00±20.86	Anoxic 2; vascular 3	8.00±3.16	Sham stimulation	0
Cheng 2024	Parallel RCT	Active tDCS	14	11/3	44.21±15.49	MCS 5; UWS/VS 9	1.73±0.69	TBI 7; hemorrhagic 6; anoxia 1	7.00±2.75	Left DLPFC	NR
		Sham tDCS	14	11/3	46.71±14.33	MCS 4; UWS/VS 10	2.00±1.15	TBI 8; hemorrhagic 3; anoxia 3	5.29±2.73	Sham stimulation	NR
Carrière 2020	Crossover RCT	Active tDCS	3	3/0	45.67±15.31	MCS 3	11.67±11.55	TBI 2; aneurysm 1	8.67±0.58	Left DLPFC	NR
		Sham tDCS	7	4/3	47.57±16.25	MCS 7	6.71±4.42	Cardiac arrest 2; aneurysm 3; meningitis 1; TBI 1	8.86±4.88	Sham stimulation	NR
Barra 2022	Crossover RCT	Active tDCS	2	0/2	41.00±14.14	MCS 2	4.50±0.71	Stroke 1; anoxic 1	9.00±0.00	Left prefrontal cortex	0
		Sham tDCS	5	2/3	51.40±18.35	MCS 5	4.66±4.57	Stroke 3; TBI 2	9.40±4.34	Sham stimulation	0
Ma 2026	Parallel RCT	Active tDCS	11	8/3	49.45±13.90	NR	0.47±0.24	Traumatic 6; vascular 4; other 1	5.55±2.42	Left DLPFC	Skin erythema, 6 cases
		Sham tDCS	11	5/6	47.27±16.06	NR	0.49±0.20	Traumatic 1; vascular 9; other 1	5.45±2.42	Sham stimulation	0

Note: Values are presented as mean ± SD unless otherwise stated. For crossover RCTs, the active tDCS and sham tDCS groups refer to the first-period allocation. 0 indicates that no adverse events or no tDCS-related adverse events were explicitly reported.

Table E4.2. tDCS stimulation parameters of included studies

Study	Stimulation target	Anode location	Cathode/reference location	Current intensity (mA)	Session duration (min)	Number of sessions	Total stimulation time (min)	Electrode area (cm²)	Current density (mA/cm²)	Cumulative dose (mA·min)
Zhang 2017	Left DLPFC	Left DLPFC, F3	Right supraorbital area, approximately Fp2	2	20	20	400	35	0.057	800
Yu 2022	Left DLPFC	Left DLPFC, F3	Right supraorbital area, Fp2	2	20	10	200	35	0.057	400
Wu 2019-A	Left DLPFC	Left DLPFC, F3	Right supraorbital area, Fp2	2	20	10	200	35	0.057	400
Wu 2019-B	Right DLPFC	Right DLPFC, F4	Contralateral supraorbital area, Fp1	2	20	10	200	35	0.057	400
Thibaut 2017	Left DLPFC	Left DLPFC, F3	Right supraorbital area	2	20	5	100	35	0.057	200
Thibaut 2023	Left DLPFC	Left DLPFC, F3	Right supraorbital area, Fp2	2	20	20	400	25	0.080	800
Martens 2018	Left DLPFC	Left DLPFC, F3	Right supraorbital area	2	20	20	400	35	0.057	800
Martens 2020	Bilateral frontoparietal network	F3, F4, CP5, CP6	Fp2, Fpz, O1, Oz	1 per anode; total current 4 per session	20	1	20	3.14 per electrode	0.318 per anode	80
Martens 2019	Primary motor cortex (M1)	C3 or C4, over the most affected motor cortex	Contralateral supraorbital area	2	20	1	20	35	0.057	40
Huang 2017	Posterior parietal cortex	Posterior parietal cortex, Pz	Right supraorbital area	2	20	5	100	NR	NR	200
Estraneo 2017	Left DLPFC	Left DLPFC, F3	Right supraorbital area, Fp2	2	20	5	100	35	0.057	200
Cheng 2024	Left DLPFC	Left DLPFC, F3	Right shoulder	2	20	20	400	35	0.057	800
Carrière 2020	Left DLPFC	Left DLPFC, F3	Right supraorbital area	2	20	1	20	35	0.057	40
Barra 2022	Left prefrontal cortex	F3	Contralateral orbitofrontal area	2	20	1	20	12	0.167	40
Ma 2026	Left DLPFC	Left DLPFC, F3	Right supraorbital area, Fp2	2	20	20	400	35	0.057	800

Note: DLPFC, dorsolateral prefrontal cortex; M1, primary motor cortex; NR, not reported. Cumulative dose was calculated as current intensity × session duration × number of sessions.

Appendix E5. Risk of bias assessment

Table E5.1. RoB 2 assessment summary by domain

Study	D1	D2	D3	D4	D5	Overall RoB	Main reasons
Zhang 2017	Low	Low	Low	Low	Some concerns	Some concerns	Randomization, sham stimulation, outcome measurement and missing data were generally acceptable. Some concern remained because no trial registration, protocol, or prespecified statistical analysis plan was reported.
Yu 2022	Low	Some concerns	Low	Low	Some concerns	Some concerns	Randomization and CRS-R outcome measurement were adequate, and no important missing outcome data were identified. Some concern arose because physicians and nurses were aware of group allocation, and no full protocol or SAP was available to confirm prespecification of analyses.
Wu 2019	Some concerns	Low	Low	Low	Low	Some concerns	The main concern was insufficient reporting of random sequence generation and allocation concealment. Sham stimulation, blinded CRS-R assessment, complete CRS-R data, and registered outcome reporting reduced concerns in other domains.
Thibaut 2017	Low	Low	Some concerns	Low	Low	Some concerns	Randomization, blinding and sham procedures were adequate; the main concern was post-randomization attrition with potentially clinically related missingness.
Thibaut 2023	Low	Low	Low	Low	Low	Low	This registered multicenter triple-blind sham-controlled trial used adequate randomization, allocation concealment, blinding, standardized CRS-R assessment, and intention-to-treat analysis. Missing data were balanced and unlikely to materially bias the result.
Martens 2018	Low	Low	Low	Low	Low	Low	The trial used computer-generated randomization, third-party allocation, blinding of patients/caregivers/outcome assessors, credible sham stimulation, and complete individual CRS-R data for the modified ITT sample. Missing data were unlikely to materially bias the first-period result.
Martens 2020	Low	Low	Low	Low	Low	Low	Randomization and blinding were adequately implemented, sham stimulation was credible, all randomized participants had behavioral CRS-R data, and the CRS-R analysis was clearly specified with complete individual data reporting.

Martens 2019	Low	Low	Low	Low	Some concerns	Some concerns	Blinding, sham stimulation, CRS-R measurement and completeness of outcome data were adequate. Some concern remained because no trial registration, protocol, or SAP was reported, making prespecification of the CRS-R analysis unclear.
Huang 2017	Some concerns	Low	Some concerns	Low	Low	Some concerns	The study was registered and used blinded CRS-R assessment with stable co-interventions. Concerns arose because random sequence generation and allocation concealment were insufficiently described, and 4 randomized participants did not complete the study with limited information on dropout distribution.
Estraneo 2017	Some concerns	Low	Some concerns	Low	Some concerns	Some concerns	Sham stimulation, blinding and CRS-R measurement were generally adequate. Concerns arose from unclear random sequence generation and allocation concealment, exclusion of 2 patients due to acute medical complications, and absence of trial registration or SAP.
Cheng 2024	Low	Low	Some concerns	Low	Low	Some concerns	Randomization, blinding, sham stimulation and CRS-R measurement were adequate. Some concern arose because 5 participants withdrew after randomization, all from the active group, and information on their baseline prognosis or relation to outcome was limited.
Carrière 2020	Low	Low	Some concerns	Low	Low	Some concerns	Blinding, sham stimulation, CRS-R measurement and reporting were generally adequate. Some concern remained because several participants were excluded due to missing behavioral data, with limited information on the reasons and baseline comparability of excluded patients.
Barra 2022	Low	Low	Low	Low	Low	Low	The registered double-blind crossover trial used sealed randomization envelopes, credible sham stimulation, blinded patients/treatment administrators/outcome assessors, standardized CRS-R assessment, and complete first-period CRS-R data for the tDCS versus sham comparison.
Ma 2026	Low	Low	Low	Low	Low	Low	The prospectively registered trial used computer-generated randomization, opaque sealed envelopes for allocation concealment, blinded participants and outcome assessors, credible sham stimulation, complete follow-up, and prespecified CRS-R assessment at fixed time points.

Abbreviations: D1, bias arising from the randomization process; D2, bias due to deviations from intended interventions; D3, bias due to missing outcome data; D4, bias in measurement of the outcome; D5, bias in selection of the reported result; RoB, risk of bias; CRS-R, Coma Recovery Scale-Revised; tDCS, transcranial direct current stimulation; SAP, statistical analysis plan; ITT, intention-to-treat.

Appendix E6. Subgroup analyses for CRS-R change scores

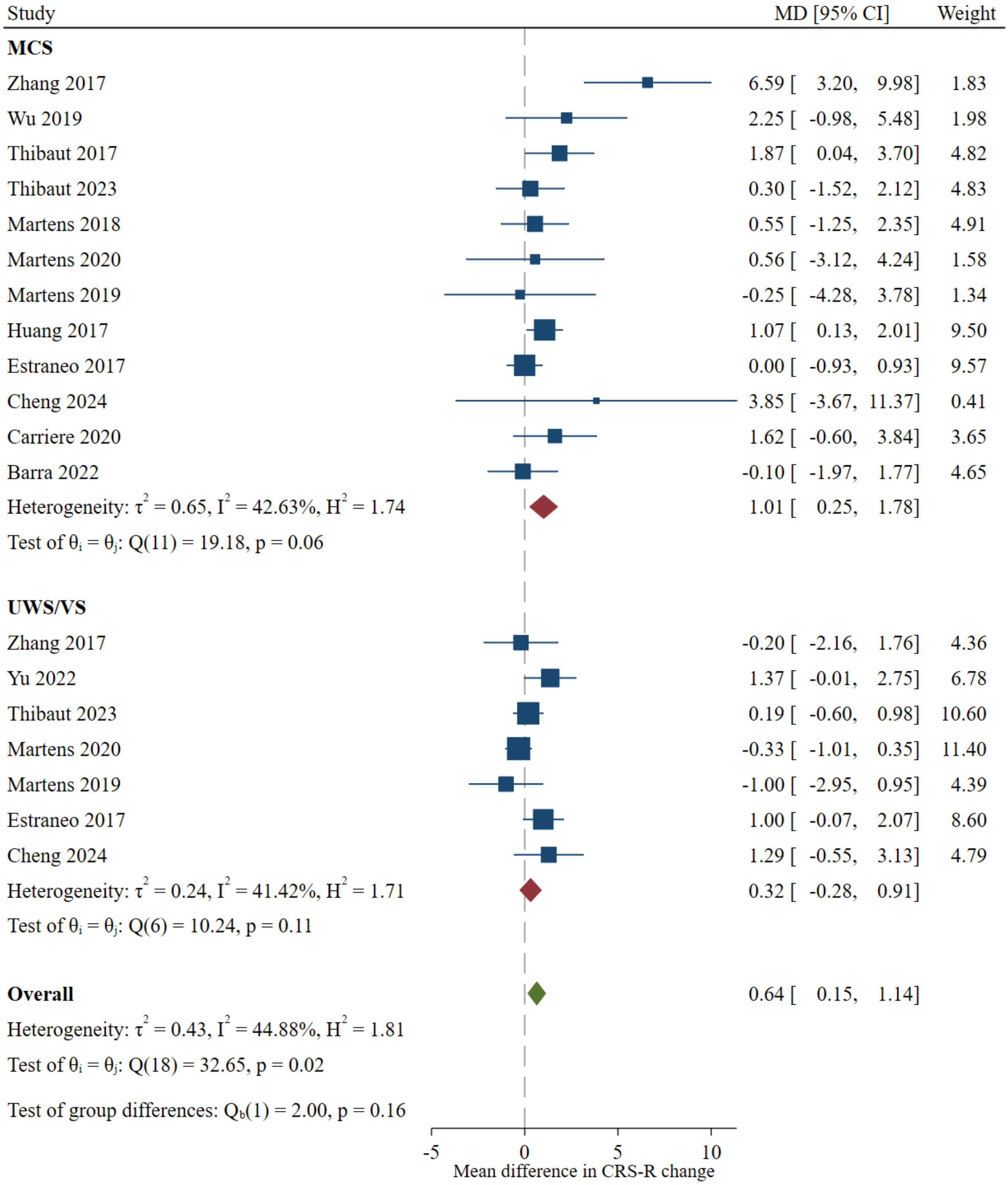

Figures E6.1. Forest plot stratified by baseline consciousness level

Note: Forest plot of the subgroup analysis stratified by baseline consciousness level. The effect size was expressed as mean difference in CRS-R change scores between active tDCS and sham stimulation. MD > 0 indicates greater improvement in the active tDCS group.

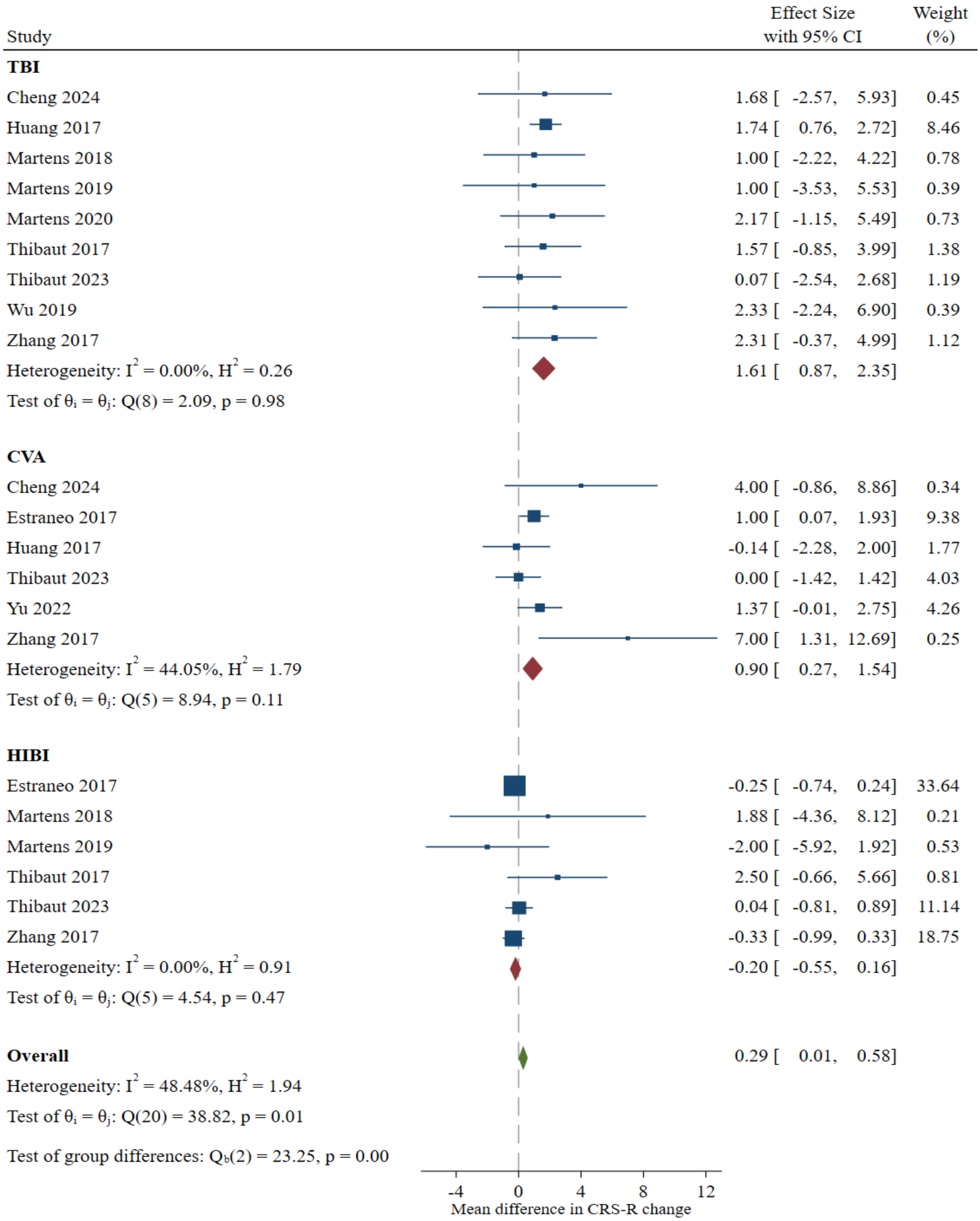

Figures E6.2. Forest plot stratified by etiology

Note: Forest plot of the subgroup analysis stratified by etiology, including traumatic brain injury (TBI), cerebrovascular accident (CVA), and hypoxic-ischemic brain injury (HIBI). The effect size was expressed as mean difference in CRS-R change scores between active tDCS and sham stimulation. MD > 0 indicates greater improvement in the active tDCS group.

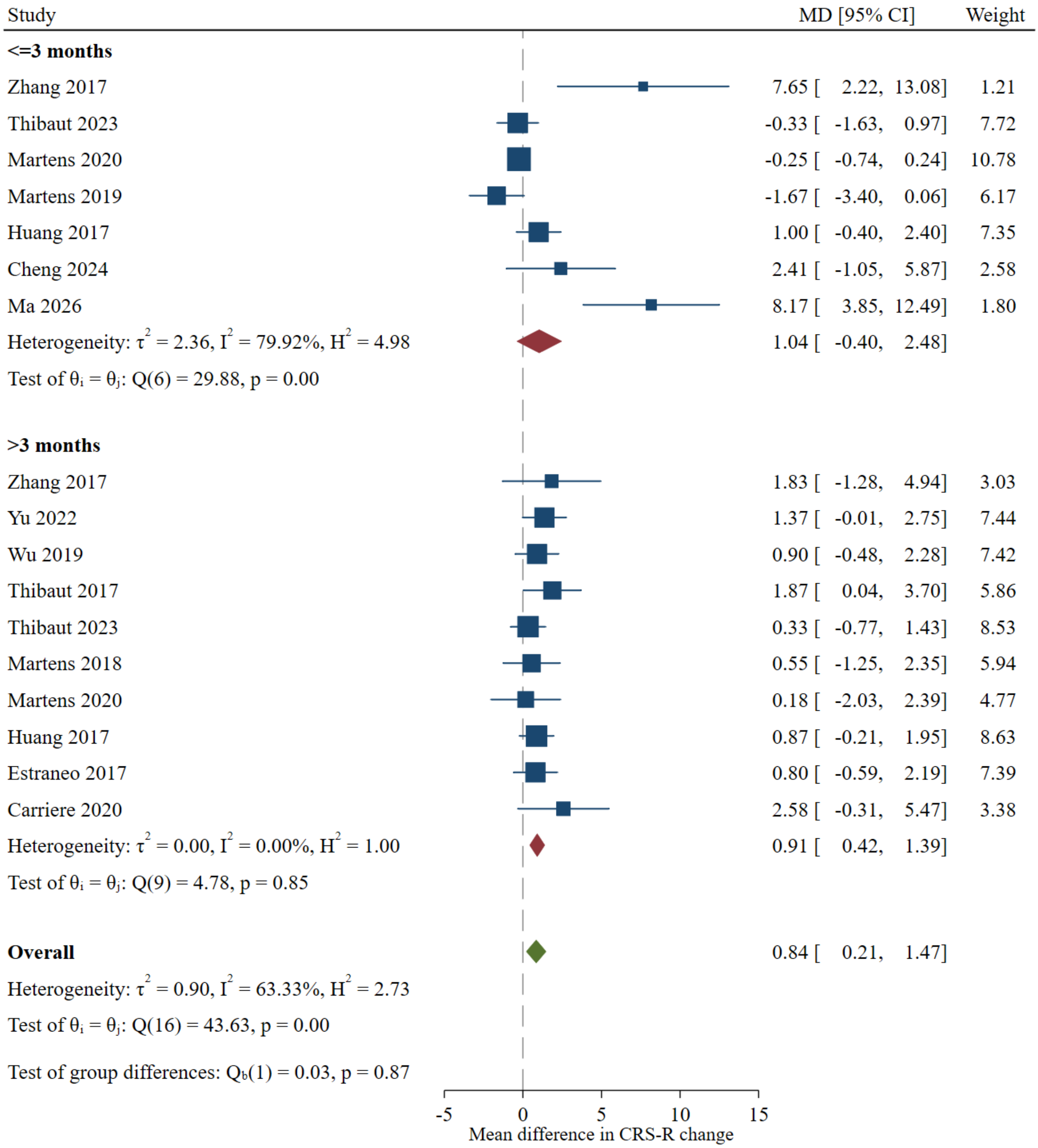

Figures E6.3. Forest plot stratified by disease duration

Note: Forest plot of the subgroup analysis stratified by disease duration, comparing patients with disease duration ≤3 months and >3 months. The effect size was expressed as mean difference in CRS-R change scores between active tDCS and sham stimulation. MD > 0 indicates greater improvement in the active tDCS group.

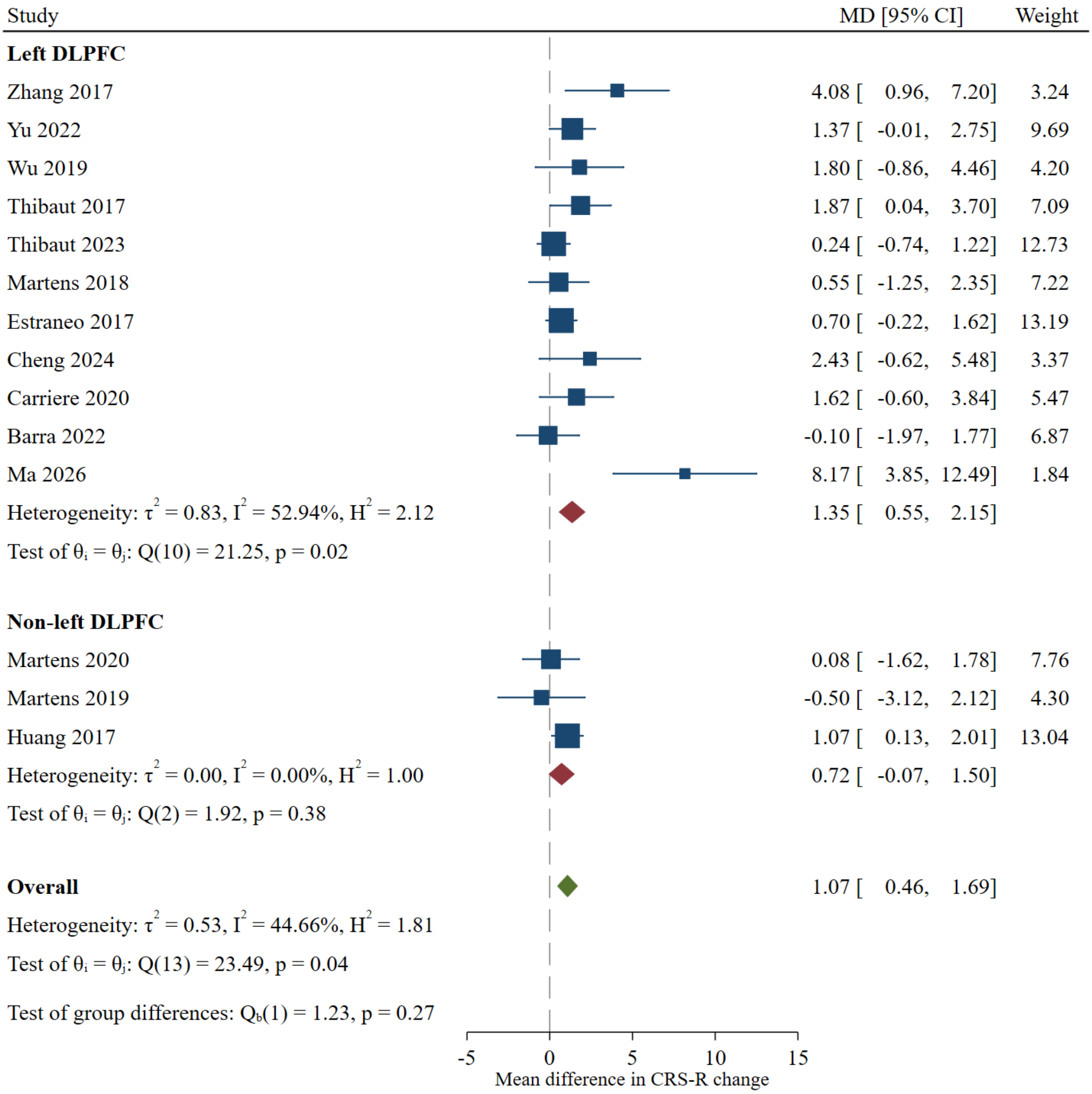

Figures E6.4. Forest plot stratified by stimulation target

Note: Forest plot of the subgroup analysis stratified by stimulation target, comparing left DLPFC and non-left DLPFC stimulation. The effect size was expressed as mean difference in CRS-R change scores between active tDCS and sham stimulation. MD > 0 indicates greater improvement in the active tDCS group.

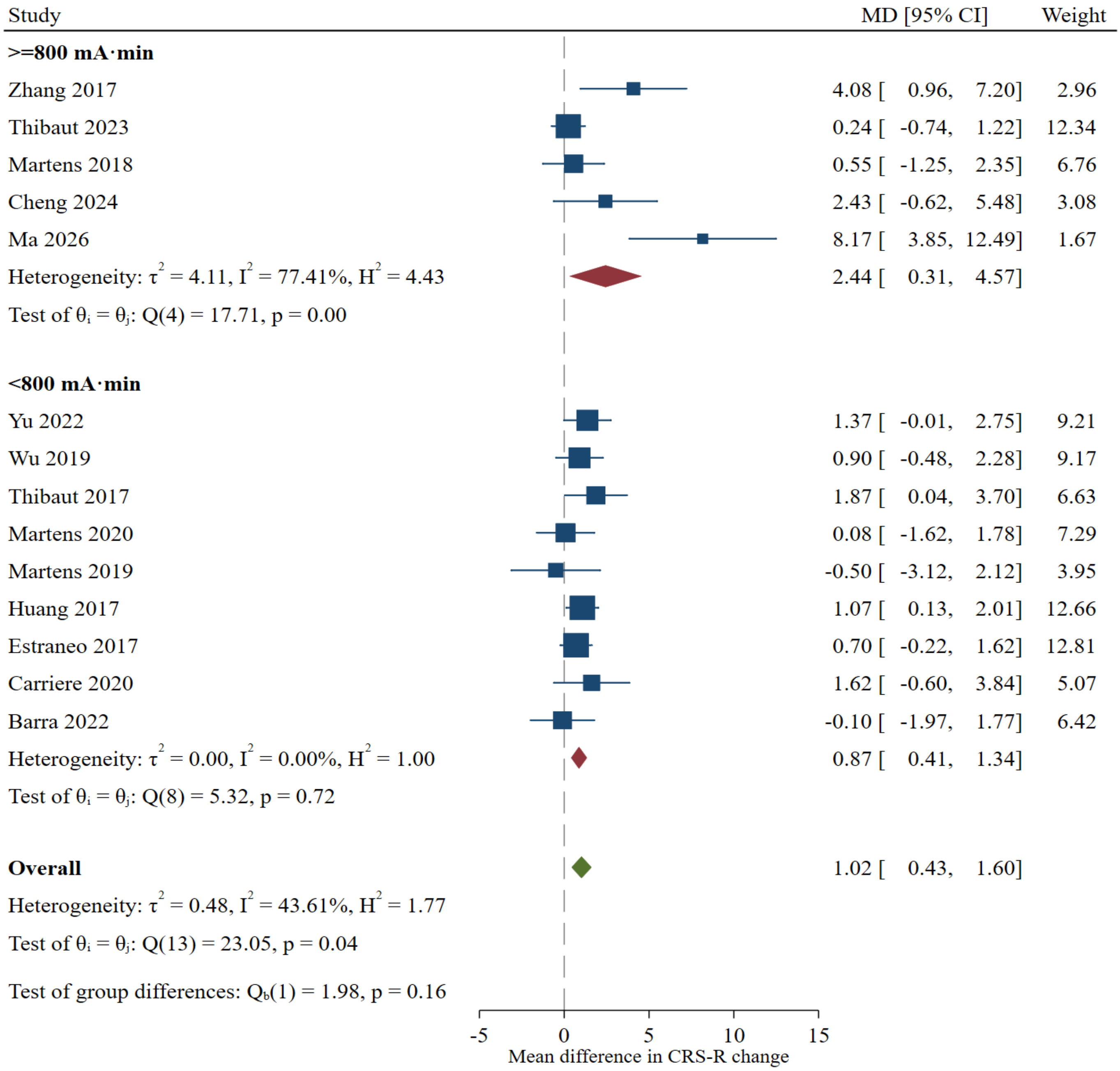

Figures E6.5. Forest plot stratified by total stimulation dose

Note: Forest plot of the subgroup analysis stratified by total stimulation dose, comparing ≥800 mA·min and <800 mA·min. The effect size was expressed as mean difference in CRS-R change scores between active tDCS and sham stimulation. MD > 0 indicates greater improvement in the active tDCS group.

Table E6.1. Sensitivity analysis for stimulation-target classification

Analysis	Subgroup	No.	MD (95% CI)	I²	P for interaction
Barra 2022 classified as left DLPFC	Left DLPFC	11	1.35 (0.55–2.15)	52.94%	0.27
	Non-left DLPFC	3	0.72 (−0.07–1.50)	0.00%	
Barra 2022 classified as non-left DLPFC	Left DLPFC	10	1.52 (0.66–2.37)	54.69%	0.11
	Non-left DLPFC	4	0.60 (−0.13–1.32)	0.00%	

Note: This table presents the sensitivity analysis in which Barra 2022 was reclassified from the left DLPFC subgroup to the non-left DLPFC subgroup.

Appendix E7. Sensitivity analyses for the primary outcome

Table E7.1. Leave-one-out sensitivity analysis for CRS-R change scores

Omitted study	No.	MD (95% CI)	P value	I^2 (%)	τ^2
Zhang 2017	13	0.91 (0.36–1.46)	0.0012	36.79	0.337
Yu 2022	13	1.00 (0.36–1.64)	0.0021	46.77	0.554
Wu 2019	13	1.05 (0.41–1.70)	0.0014	47.94	0.580
Thibaut 2017	13	0.96 (0.35–1.57)	0.0021	45.19	0.497
Thibaut 2023	13	1.14 (0.50–1.77)	0.0005	42.84	0.519
Martens 2018	13	1.07 (0.44–1.70)	0.0009	47.62	0.549
Martens 2020	13	1.10 (0.48–1.72)	0.0005	45.75	0.513
Martens 2019	13	1.08 (0.48–1.68)	0.0004	45.31	0.484
Huang 2017	13	1.04 (0.37–1.71)	0.0023	47.56	0.637
Estraneo 2017	13	1.09 (0.42–1.77)	0.0014	47.48	0.639
Cheng 2024	13	0.98 (0.38–1.57)	0.0014	45.59	0.486
Carrière 2020	13	0.99 (0.38–1.61)	0.0015	46.96	0.524
Barra 2022	13	1.10 (0.49–1.71)	0.0004	45.27	0.497
Ma 2026	13	0.83 (0.43–1.23)	0.0001	0.53	0.003

Note: MD > 0 indicates greater improvement in CRS-R change scores in the active tDCS group than in the sham tDCS group. τ^2 represents the between-study variance.

Table E7.2. Summary of sensitivity analyses for the effect of active tDCS on CRS-R change scores

Sensitivity analysis	Excluded studies	No.	MD (95% CI)	P value	Interpretation
Primary analysis	None	14	1.02 (0.43–1.60)	<0.001	Active tDCS significantly improved CRS-R change scores compared with sham tDCS.
Leave-one-out analysis	One study omitted at a time	13 each	0.83–1.14	0.0001–0.0023	The pooled effect remained significant after omission of any single study.
Excluding single-session tDCS studies	Martens 2020, Martens 2019, Carrière 2020, Barra 2022	10	1.28 (0.57–1.99)	<0.001	The effect remained significant after restricting the analysis to repeated-session tDCS studies.
Excluding very small studies/comparisons	Martens 2019, Carrière 2020, Barra 2022	11	1.16 (0.50–1.83)	<0.001	The effect was not driven by very small studies/comparisons.
Excluding studies with derived change-score SDs	Ma 2026	13	0.83 (0.43–1.23)	<0.001	The effect remained significant, and heterogeneity was markedly reduced.

Notes: MD > 0 indicates greater improvement in CRS-R change scores in the active tDCS group than in the sham tDCS group. Very small studies/comparisons were defined as studies/comparisons with a total sample size ≤10 or with fewer than five participants in either treatment arm. Derived change-score SD refers to change-score SD calculated from baseline and post-intervention SDs using an assumed pre–post correlation coefficient.

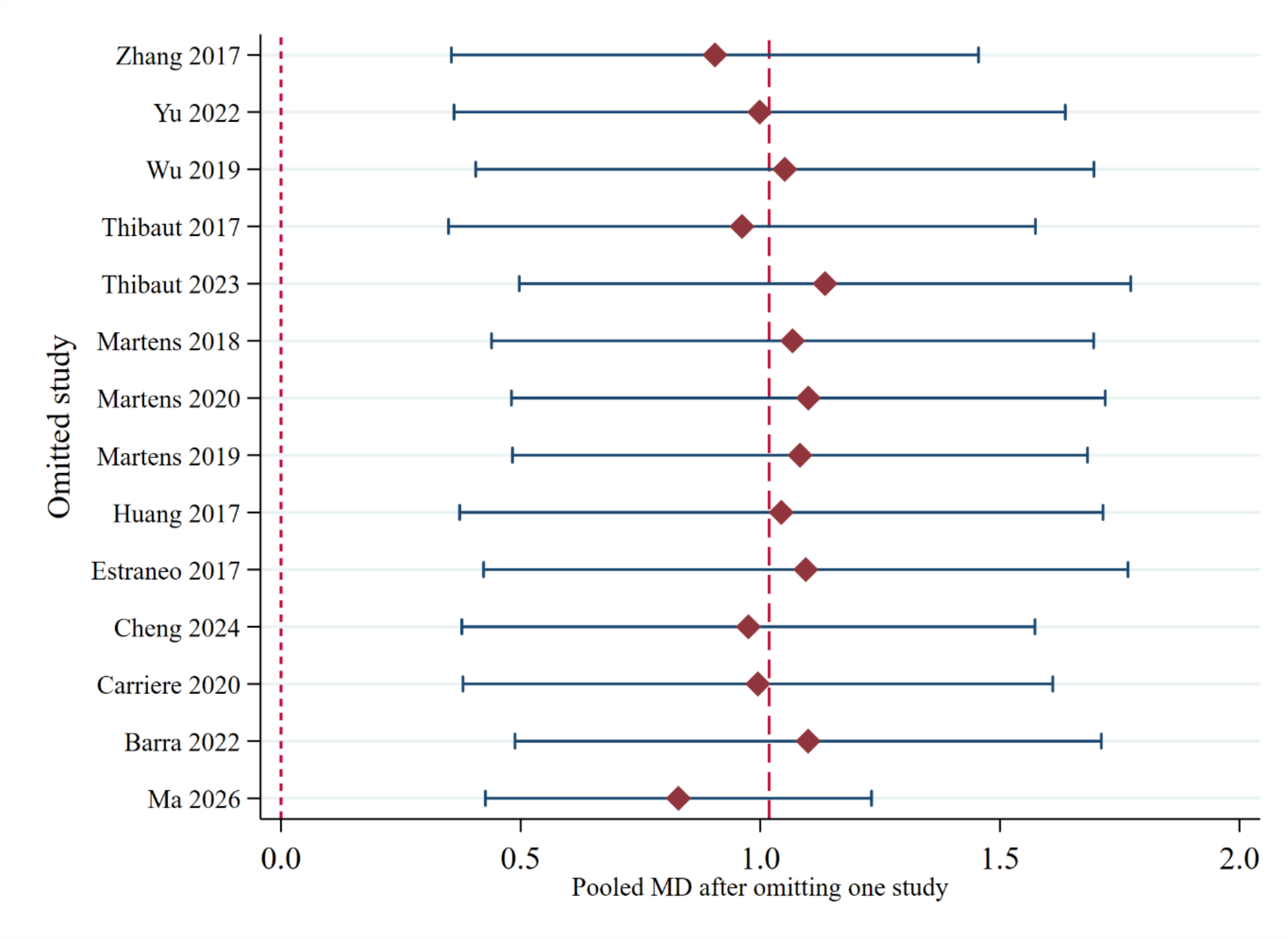

Figures E7.1. Leave-one-out sensitivity analysis for CRS-R change scores

Notes: This figure shows the pooled mean difference after sequential omission of each included study. The pooled effect remained statistically significant after excluding any single study.

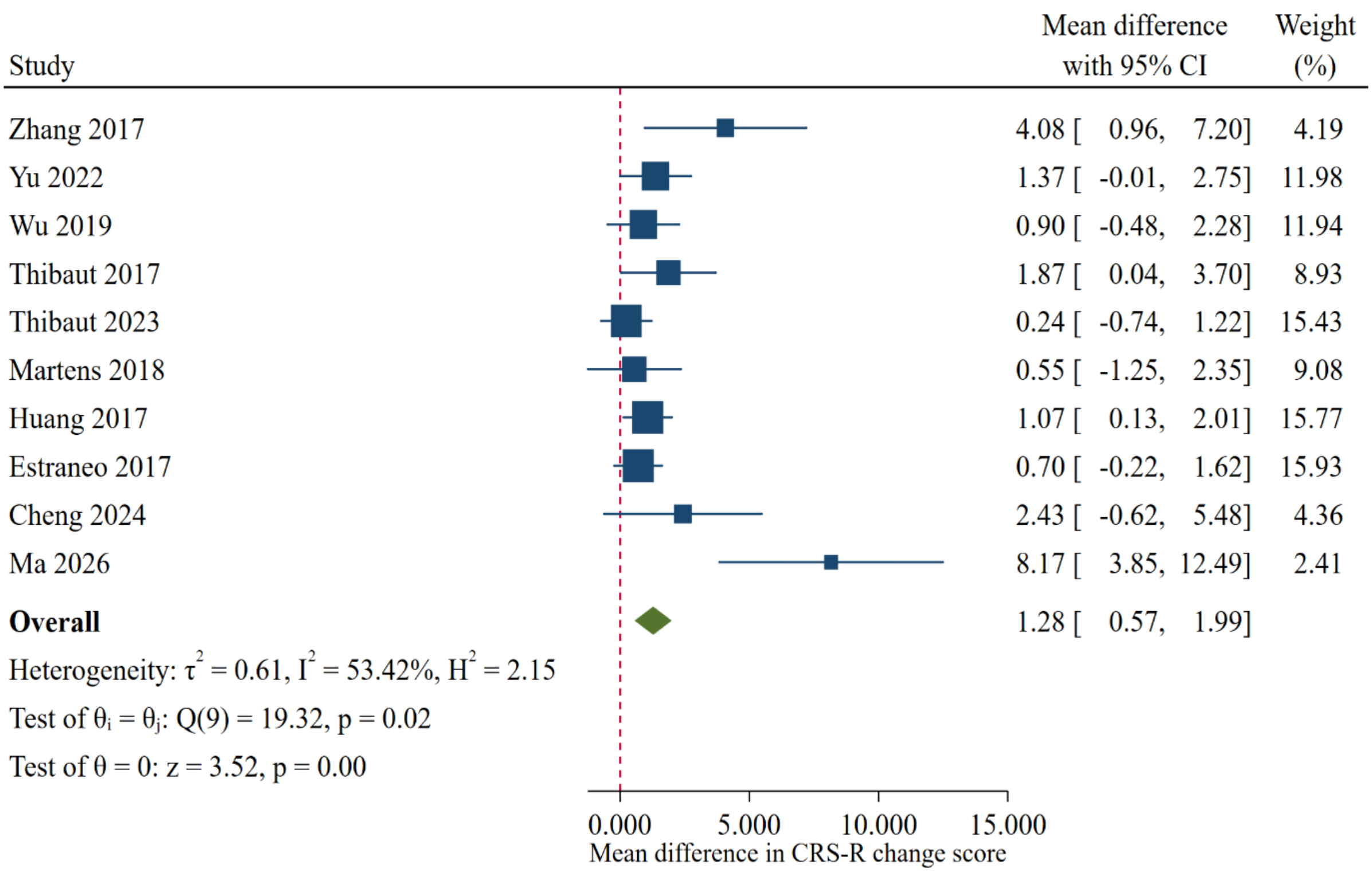

Figures E7.2. Sensitivity analysis excluding single-session tDCS studies

Notes: This forest plot shows the pooled effect after excluding single-session tDCS studies. The result remained statistically significant in favor of active tDCS.

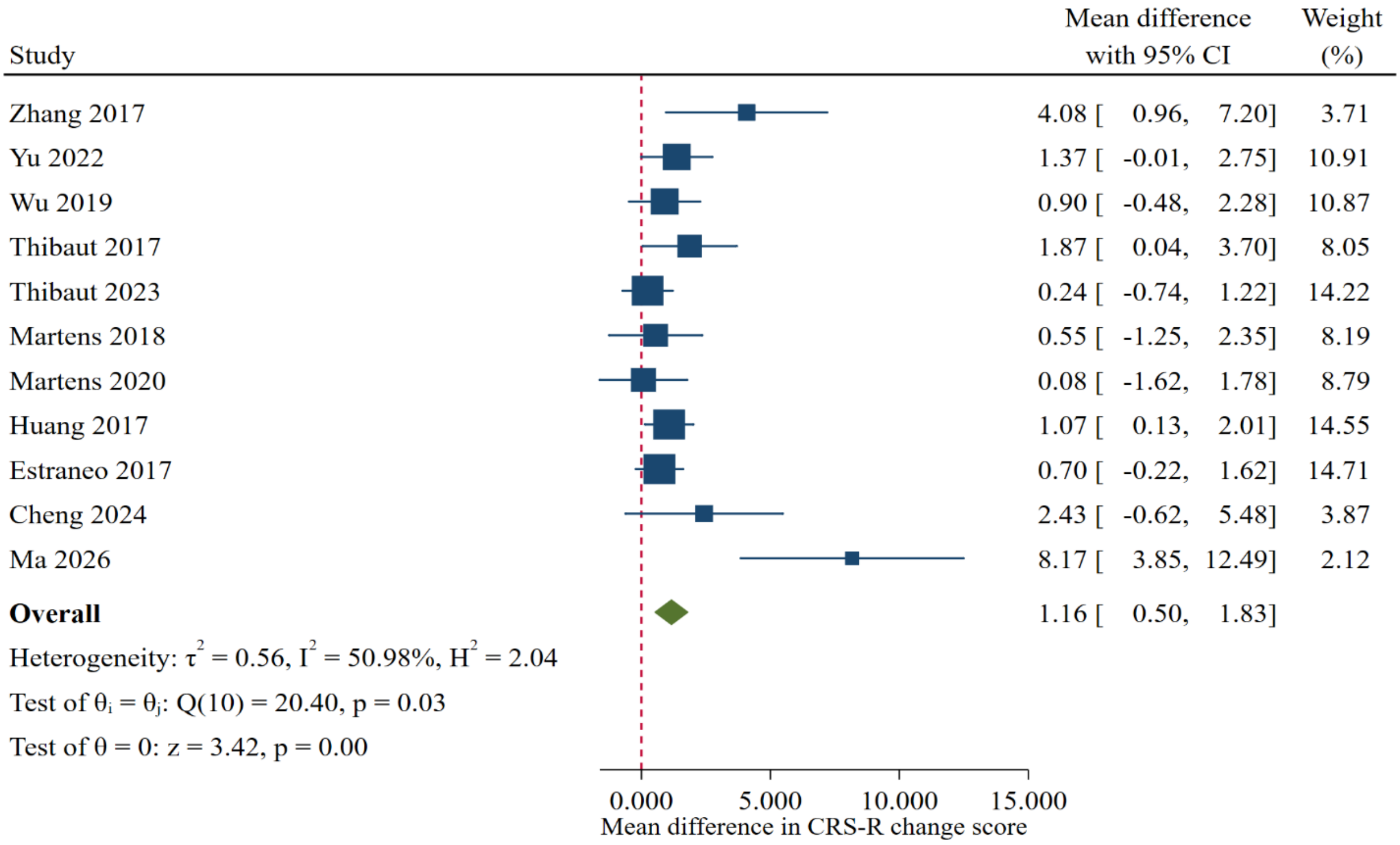

Figures E7.3. Sensitivity analysis excluding very small studies or comparisons

Notes: This forest plot shows the pooled effect after excluding studies/comparisons with a total sample size ≤10 or fewer than five participants in either treatment arm. The result remained statistically significant.

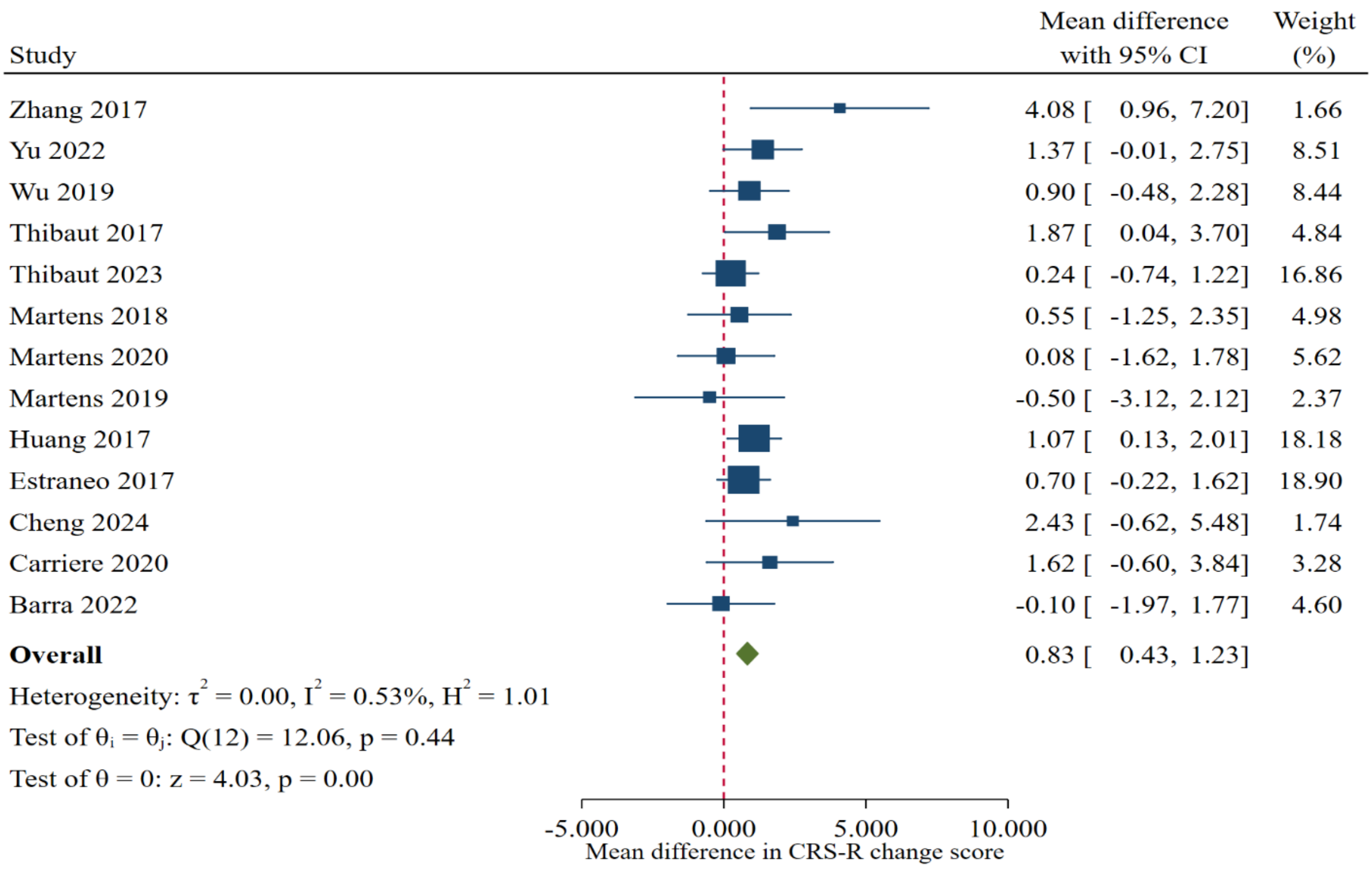

Figures E7.4. Sensitivity analysis excluding studies with derived change-score SDs

Notes: This forest plot shows the pooled effect after excluding Ma 2026, for which the change-score SD was derived using an assumed pre–post correlation coefficient. The pooled effect remained statistically significant, and between-study heterogeneity was markedly reduced.

Appendix E8. Publication bias and small-study effects for the primary analysis

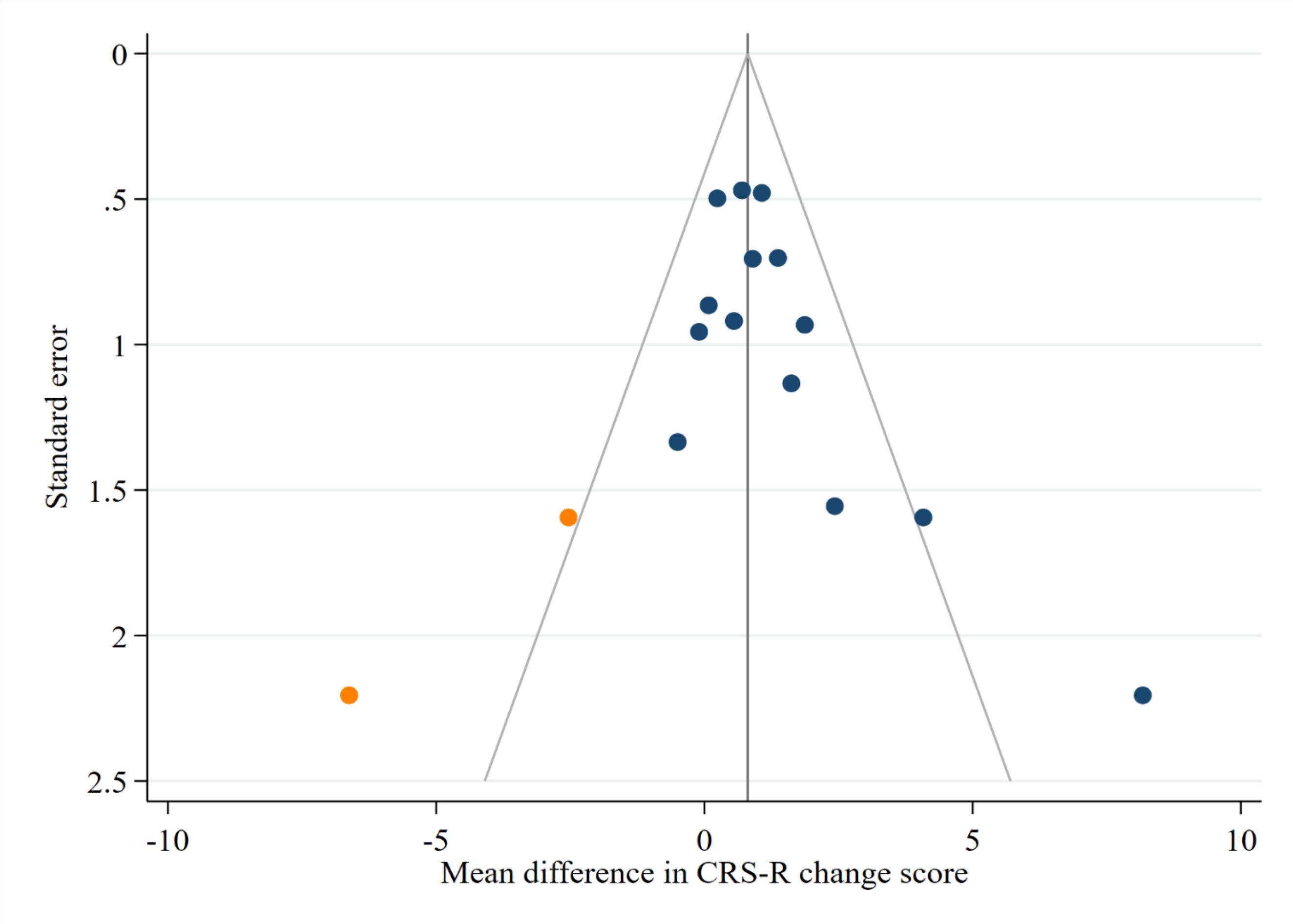

Figures E8.1. Trim-and-fill funnel plot for the primary analysis of CRS-R change scores

Note: The trim-and-fill analysis was performed to explore the potential influence of publication bias or small-study effects on the primary analysis. Two potentially missing studies were imputed on the left side of the funnel plot. After imputation, the pooled effect was attenuated but remained statistically significant. Blue circles indicate observed studies, and orange circles indicate imputed studies.

Table E8.1. Tests for publication bias and small-study effects in the primary analysis

Test	Statistic	Standard error	z value	P value
Egger's regression test	β = 1.94	0.749	2.59	0.0095
Begg's rank correlation test	Kendall's score = 33.00	18.267	1.75	0.0798

Note: Egger's regression test and Begg's rank correlation test were used to assess potential small-study effects or publication bias in the primary analysis. The null hypothesis was no small-study effects or funnel plot asymmetry. A P value <0.05 was considered statistically significant.

Table E8.2. Trim-and-fill analysis for the primary analysis of CRS-R change scores

Analysis	No. of studies	No. of imputed studies	Pooled MD	95% CI
Observed studies	14	0	1.018	0.433–1.604
Observed + imputed studies	16	2	0.806	0.091–1.521

Note: The trim-and-fill analysis estimated that two potentially missing studies were imputed on the left side. MD represents the mean difference in CRS-R change scores between active tDCS and sham tDCS.

Appendix E9. GRADE summary of findings and certainty of evidence

Table E9.1. Summary of findings and GRADE assessment of the certainty of evidence

Active transcranial direct current stimulation compared with sham stimulation for patients with disorders of consciousness

Patient or population: patients with disorders of consciousness

Settings: acute, subacute, or chronic disorders of consciousness

Intervention: active transcranial direct current stimulation

Comparison: sham transcranial direct current stimulation

Outcomes	Illustrative comparative risks* (95% CI)		Relative effect (95% CI)	No of Participants (studies)	Certainty of the evidence (GRADE)	Comments
	Assumed risk	Corresponding risk				
	**Sham transcranial direct current stimulation**	**Active transcranial direct current stimulation**				
**CRS-R change score** Coma Recovery Scale-Revised change score from baseline Follow-up: 0–4 weeks	Not applicable.	The mean CRS-R change score in the active tDCS group was 1.02 points higher than that in the sham tDCS group (95% CI 0.43 to 1.60 higher).		330 (14 studies)	⊕⊕⊖⊖ **low**[1,2,3,4,5]	
**Adverse events** Reported adverse events during or immediately after intervention Follow-up: 0–4 weeks	Not applicable.	No pooled estimate was calculated. No clear tDCS-related serious adverse events were reported; mild and transient adverse events were reported in some studies.	Not pooled; no effect estimate was calculated	14 studies included; 11 provided adverse-event information	⊕⊖⊖⊖ **very low**[6,7,8,9]	

CI: confidence interval; CRS-R: Coma Recovery Scale-Revised; DoC: disorders of consciousness; GRADE: Grading of Recommendations Assessment, Development and Evaluation; MD: mean difference; tDCS: transcranial direct current stimulation.

[1] Risk of bias: downgraded one level because 9 of 14 studies had some concerns, although none was judged at high risk of bias.

[2] Inconsistency: not downgraded because heterogeneity was moderate (I^2 = 43.61%) and the direction of effect remained stable across sensitivity analyses.

[3] Indirectness: not downgraded because the population, intervention, comparator, and outcome directly addressed the review question.

[4] Imprecision: not downgraded because the certainty assessment concerned the presence of a non-null average effect on CRS-R change; the pooled MD was 1.02 (95% CI 0.43–1.60), and the confidence interval did not cross the null. Wide subgroup CIs were considered limitations of the exploratory subgroup analyses rather than of the overall pooled estimate.

[5] Publication bias: downgraded one level because funnel plot asymmetry and Egger's test suggested possible publication bias or small-study effects, and trim-and-fill attenuated the pooled estimate. These findings were considered suggestive rather than conclusive because only 14 studies were available.

[6] Downgraded one level for risk of bias because adverse events were not systematically assessed or consistently reported across studies.

[7] Downgraded one level for inconsistency because definitions, recording methods, and group-specific reporting of adverse events varied across studies.

[8] Downgraded two levels for imprecision because the total sample size was small, adverse events were sparse, and no pooled estimate could be calculated.

[9] Downgraded one level for reporting bias because 3 of the 14 included studies did not provide extractable group-specific safety data.

Appendix E10. References and analysis inclusion status of included studies

E10.1 References of included randomized controlled trials

(1) Zhang Y, Song W, Du J, Huo S, Shan G, Li R. Transcranial Direct Current Stimulation in Patients with Prolonged Disorders of Consciousness: Combined Behavioral and Event-Related Potential Evidence. Front Neurol. 2017 Nov 21;8:620. doi: 10.3389/fneur.2017.00620. PMID: 29209270; PMCID: PMC5702306.

(2) Yu J, Wu Y, Wu B, Xu C, Cai J, Wen X, Meng F, Zhang L, He F, Hong L, Gao J, Li J, Yu J, Luo B. Sleep patterns correlates with the efficacy of tDCS on post-stroke patients with prolonged disorders of consciousness. J Transl Med. 2022 Dec 15;20(1):601. doi: 10.1186/s12967-022-03710-2. PMID: 36522680; PMCID: PMC9756665.

(3) Wu M, Yu Y, Luo L, Wu Y, Gao J, Ye X, Luo B. Efficiency of Repetitive Transcranial Direct Current Stimulation of the Dorsolateral Prefrontal Cortex in Disorders of Consciousness: A Randomized Sham-Controlled Study. Neural Plast. 2019 Jun 11;2019:7089543. doi: 10.1155/2019/7089543. PMID: 31308848; PMCID: PMC6594311.

(4) Thibaut A, Wannez S, Donneau AF, Chatelle C, Gosseries O, Bruno MA, Laureys S. Controlled clinical trial of repeated prefrontal tDCS in patients with chronic minimally conscious state. Brain Inj. 2017;31(4):466-474. doi: 10.1080/02699052.2016.1274776. Epub 2017 Mar 10. PMID: 28281845.

(5) Thibaut A, Fregni F, Estraneo A, Fiorenza S, Noe E, Llorens R, Ferri J, Formisano R, Morone G, Bender A, Rosenfelder M, Lamberti G, Kodratyeva E, Kondratyev S, Legostaeva L, Suponeva N, Krewer C, Müller F, Dardenne N, Jedidi H, Laureys S, Gosseries O, Lejeune N, Martens G; IBIA DOC-SIG. Sham-controlled randomized multicentre trial of transcranial direct current stimulation for prolonged disorders of consciousness. Eur J Neurol. 2023 Oct;30(10):3016-3031. doi: 10.1111/ene.15974. Epub 2023 Jul 28. PMID: 37515394.

(6) Martens G, Lejeune N, O'Brien AT, Fregni F, Martial C, Wannez S, Laureys S, Thibaut A. Randomized controlled trial of home-based 4-week tDCS in chronic minimally conscious state. Brain Stimul. 2018 Sep-Oct;11(5):982-990. doi: 10.1016/j.brs.2018.04.021. Epub 2018 May 2. PMID: 29759943.

(7) Martens G, Kroupi E, Bodien Y, Frasso G, Annen J, Cassol H, Barra A, Martial C, Gosseries O, Lejeune N, Soria-Frisch A, Ruffini G, Laureys S, Thibaut A. Behavioral and electrophysiological effects of network-based frontoparietal tDCS in patients with severe brain injury: A randomized controlled trial. Neuroimage Clin. 2020;28:102426. doi: 10.1016/j.nicl.2020.102426. Epub 2020 Sep 15. PMID: 32977212; PMCID: PMC7511767.

(8) Martens G, Fregni F, Carrière M, Barra A, Laureys S, Thibaut A. Single tDCS session of motor cortex in patients with disorders of consciousness: a pilot study. Brain Inj. 2019;33(13-14):1679-1683. doi: 10.1080/02699052.2019.1667537. Epub 2019 Sep 15. PMID: 31523995.

(9) Huang W, Wannez S, Fregni F, Hu X, Jing S, Martens G, He M, Di H, Laureys S, Thibaut A. Repeated stimulation of the posterior parietal cortex in patients in minimally conscious state: A sham-controlled randomized clinical trial. Brain Stimul. 2017 May-Jun;10(3):718-720. doi: 10.1016/j.brs.2017.02.001. Epub 2017 Feb 16. PMID: 28259543.

(10) Estraneo A, Pascarella A, Moretta P, Masotta O, Fiorenza S, Chirico G, Crispino E, Loreto V, Trojano L. Repeated transcranial direct current stimulation in prolonged disorders of consciousness: A double-blind cross-over study. J Neurol Sci. 2017 Apr 15;375:464-470. doi: 10.1016/j.jns.2017.02.036. Epub 2017 Feb 17. PMID: 28320187.

(11) Cheng XR, Zhang YB, Sun DJ, Peng XY, Bao YC, Zhang F, Wang MX. Long-term repetitive transcranial direct current stimulation in patients with disorders of consciousness: a preliminary study. Brain Inj. 2024 Jan 28;38(2):68-75. doi: 10.1080/02699052.2024.2304872. Epub 2024 Feb 8. PMID: 38329075.

(12) Carrière M, Mortaheb S, Raimondo F, Annen J, Barra A, Binda Fossati MC, Chatelle C, Hermann B, Martens G, Di Perri C, Laureys S, Thibaut A. Neurophysiological Correlates of a Single Session of Prefrontal tDCS in Patients with Prolonged Disorders of Consciousness: A Pilot Double-Blind Randomized Controlled Study. Brain Sci. 2020 Jul 21;10(7):469. doi: 10.3390/brainsci10070469. PMID: 32708119; PMCID: PMC7408434.

(13) Barra A, Rosenfelder M, Mortaheb S, Carrière M, Martens G, Bodien YG, Morales-Quezada L, Bender A, Laureys S, Thibaut A, Fregni F. Transcranial Pulsed-Current Stimulation versus Transcranial Direct Current Stimulation in Patients with Disorders of Consciousness: A Pilot, Sham-Controlled Cross-Over Double-Blind Study. Brain Sci. 2022 Mar 24;12(4):429. doi: 10.3390/brainsci12040429. PMID: 35447961; PMCID: PMC9031379.

(14) Ma H, Zhao K, Hu C, Huang C, Hu X, Zhang Z, Chen X, Jia C. Repeated tDCS of the left dorsolateral prefrontal cortex for acute DoC: a pilot randomized controlled trial. Sci Rep. 2026 May 25. doi: 10.1038/s41598-026-55060-4. Epub ahead of print. PMID: 42185619

Table E10.1. Inclusion status of included studies across outcome and supplementary analyses

Study	Main analysis	Consciousness-level subgroup	Etiology subgroup	Disease-duration subgroup	Stimulation-target subgroup	Stimulation-dose subgroup	Remarks
Zhang 2017	Yes	Yes	Yes	Yes	Yes	Yes	Consciousness level: UWS/VS and MCS; etiology: TBI, CVA, and HIBI; disease duration: ≤3 months and >3 months; target: left DLPFC; dose: ≥800 mA·min.
Yu 2022	Yes	Yes	Yes	Yes	Yes	Yes	Consciousness level: UWS/VS; etiology: CVA; disease duration: >3 months; target: left DLPFC; dose: <800 mA·min.
Wu 2019	Yes	Yes	Yes	Yes	Yes	Yes	Consciousness level: UWS/VS and MCS; etiology: TBI was included, whereas CVA/HIBI were not pooled because one arm had no available data; disease duration: >3 months; target: only the left-DLPFC comparison was included; the right-DLPFC comparison was not included; dose: <800 mA·min.
Thibaut 2017	Yes	Yes	Yes	Yes	Yes	Yes	Consciousness level: MCS; etiology: TBI and HIBI; disease duration: >3 months; target: left DLPFC; dose: <800 mA·min.
Thibaut 2023	Yes	Yes	Yes	Yes	Yes	Yes	Consciousness level: UWS/VS and MCS; etiology: TBI, CVA, and HIBI; disease duration: ≤3 months and >3 months; target: left DLPFC; dose: ≥800 mA·min.
Martens 2018	Yes	Yes	Yes	Yes	Yes	Yes	Consciousness level: MCS; etiology: TBI and HIBI, whereas CVA was not pooled because one arm had no available data; disease duration: >3 months; target: left DLPFC; dose: ≥800 mA·min.
Martens 2020	Yes	Yes	Yes	Yes	Yes	Yes	Consciousness level: UWS/VS and MCS; etiology: TBI was included, whereas other etiology-stratified data were insufficient; disease duration: ≤3 months and >3 months; target: bilateral frontoparietal network; dose: <800 mA·min.
Martens 2019	Yes	Yes	Yes	Yes	Yes	Yes	Consciousness level: UWS/VS and MCS; etiology: TBI and HIBI; disease duration: ≤3 months, whereas the >3-month stratum was not pooled because of limited data or non-estimable SD; target: primary motor cortex; dose: <800 mA·min.
Huang 2017	Yes	Yes	Yes	Yes	Yes	Yes	Consciousness level: MCS; etiology: TBI and CVA, whereas HIBI was not pooled because of limited data or non-estimable SD; disease duration: ≤3 months and >3 months; target: posterior parietal cortex; dose: <800 mA·min.
Estraneo 2017	Yes	Yes	Yes	Yes	Yes	Yes	Consciousness level: UWS/VS and MCS; etiology: CVA and HIBI, whereas TBI was not pooled because one arm had no available data; disease duration: >3 months; target: left DLPFC; dose: <800 mA·min.

Cheng 2024	Yes	Yes	Yes	Yes	Yes	Yes	Consciousness level: UWS/VS and MCS, with eMCS data excluded; etiology: TBI and CVA, whereas HIBI was not pooled because of limited data or non-estimable SD; disease duration: ≤3 months; target: left DLPFC; dose: ≥800 mA·min.
Carrière 2020	Yes	Yes	No	Yes	Yes	Yes	Consciousness level: MCS; etiology: not included because etiology-stratified data were insufficient; disease duration: >3 months; target: left DLPFC; dose: <800 mA·min.
Barra 2022	Yes	Yes	No	No	Yes	Yes	Only the first-period tDCS-versus-sham comparison was included; the tPCS arm was not included in any quantitative synthesis. Consciousness level: MCS; etiology: not included because etiology-stratified data were insufficient; disease duration: not included because duration-stratified data were insufficient; target: left prefrontal cortex; dose: <800 mA·min.
Ma 2026	Yes	No	No	Yes	Yes	Yes	Consciousness level: data suitable for UWS/VS versus MCS subgrouping were not reported; etiology: extractable etiology-stratified effect data were not reported; disease duration: ≤3 months; target: left DLPFC; dose: ≥800 mA·min.

Note: The main analysis included all studies with extractable CRS-R change-score data. For crossover trials, only first-period data were extracted. The consciousness-level subgroup analysis included only data that could be clearly stratified as UWS/VS or MCS. The etiology subgroup analysis included TBI, CVA, and HIBI. Disease duration was stratified as ≤3 months or >3 months. The stimulation-dose subgroup was stratified as cumulative dose ≥800 mA·min or <800 mA·min. Some studies were included in the main analysis but not in specific subgroup analyses because subgroup-level data were insufficient, one comparison arm had no available data, or the SD could not be estimated. In Wu 2019, the right-DLPFC comparison was not included in the stimulation-target subgroup meta-analysis because the change-score variance or standard error was not estimable from the available data.
